# Systematic gene overexpression in Candida albicans identifies a regulator of early adaptation to the mammalian gut

**DOI:** 10.1111/cmi.12890

**Published:** 2018-08-07

**Authors:** Sadri Znaidi, Lasse van Wijlick, Arturo Hernández‐Cervantes, Natacha Sertour, Jean‐Luc Desseyn, Frédéric Vincent, Ralitsa Atanassova, Valérie Gouyer, Carol A. Munro, Sophie Bachellier‐Bassi, Frédéric Dalle, Thierry Jouault, Marie‐Elisabeth Bougnoux, Christophe d'Enfert

**Affiliations:** ^1^ Institut Pasteur, INRA Unité Biologie et Pathogénicité Fongiques Paris France; ^2^ Institut Pasteur de Tunis, University of Tunis El Manar Laboratoire de Microbiologie Moléculaire, Vaccinologie et Développement Biotechnologique Tunis Tunisia; ^3^ Lille Inflammation Research International Center, UMR 995 Inserm Université Lille 2, Faculté de Médecine Lille France; ^4^ UMR 1347 Université de Bourgogne Dijon France; ^5^ Medical Research Council Centre for Medical Mycology at the University of Aberdeen, Institute of Medical Sciences University of Aberdeen Aberdeen UK; ^6^ Centre Hospitalier Universitaire Service de Parasitologie Mycologie Dijon France; ^7^ Laboratoire de Parasitologie‐Mycologie, Service de Microbiologie, Hôpital Necker‐Enfants Malades Université Paris Descartes, Faculté de Médecine Paris France

**Keywords:** Candida albicans, chromatin immunoprecipitation‐on‐chip, *CRZ2*, gastrointestinal colonisation, regulatory networks, signature‐tagged overexpression, transcriptomics

## Abstract

Candida albicans is part of the human gastrointestinal (GI) microbiota. To better understand how C. albicans efficiently establishes GI colonisation, we competitively challenged growth of 572 signature‐tagged strains (~10% genome coverage), each conditionally overexpressing a single gene, in the murine gut. We identified *CRZ2*, a transcription factor whose overexpression and deletion respectively increased and decreased early GI colonisation. Using clues from genome‐wide expression and gene‐set enrichment analyses, we found that the optimal activity of Crz2p occurs under hypoxia at 37°C, as evidenced by both phenotypic and transcriptomic analyses following *CRZ2* genetic perturbation. Consistent with early colonisation of the GI tract, we show that *CRZ2* overexpression confers resistance to acidic pH and bile salts, suggesting an adaptation to the upper sections of the gut. Genome‐wide location analyses revealed that Crz2p directly modulates the expression of many mannosyltransferase‐ and cell‐wall protein‐encoding genes, suggesting a link with cell‐wall function. We show that *CRZ2* overexpression alters cell‐wall phosphomannan abundance and increases sensitivity to tunicamycin, suggesting a role in protein glycosylation. Our study reflects the powerful use of gene overexpression as a complementary approach to gene deletion to identify relevant biological pathways involved in C. albicans interaction with the host environment.

## 1 INTRODUCTION

Candida albicans is part of the human microbiota. As a commensal, C. albicans is present within the gastrointestinal (GI) and genital tracts of healthy humans. In patients with altered immunity or those undergoing broad‐spectrum antibiotic treatment, C. albicans colonises the GI tract and may cause disease (Shankar et al., 2015; Zaborin et al., 2014). C. albicans represents the most frequently isolated species from patients with fungemia and is responsible for a significant mortality rate among intensive‐care unit patients (Delaloye & Calandra, 2014). C. albicans may also participate in the onset of the inflammatory bowel disease through disruption of the gut microbial equilibrium (i.e., dysbiosis; Sokol et al., 2017). Consequently, understanding the mechanisms of C. albicans colonisation/invasion of body niches would help in proposing new GI disease‐preventive strategies and/or targets for the development of antifungal agents.

Owing to completion of the C. albicans genome sequencing project (Braun et al., 2005), functional genomics studies in C. albicans have considerably increased over the past decade. The construction of biological resources (e.g., gene knock‐out collections) combined with the use of genomics technologies has significantly contributed to our understanding of how C. albicans expresses its pathogenicity traits. Powerful in vivo screens identified a handful number of transcription factors (TFs) with a role in GI tract colonisation (*LYS144*, *TYE7*, *ZCF8*, *ZFU2*, and *TRY4*; Bohm et al., 2017; Perez, Kumamoto, & Johnson, 2013), establishment of systemic infection (*LYS14*, *ZCF21*, *RGT1*, *SEF1*, and others; Noble et al., 2010; Perez et al., 2013; Vandeputte, Ischer, Sanglard, & Coste, 2011), or both processes (*RTG1*, *RTG3*, and *HMS1*; Perez et al., 2013; Vandeputte et al., 2011). These screens relied on competitive phenotypic profiling of signature‐tagged (i.e., barcoded) gene‐deletion mutants in mouse models of GI tract colonisation or systemic infection. Additional TFs with a role in adherence to abiotic substrates (e.g., *CRZ2*, *BCR1*, and *ACE2*; Finkel et al., 2012) or biofilm formation (e.g., *BCR1* and *ROB1*; Nobile et al., 2012; Nobile & Mitchell, 2005) have been identified using in vitro screens of TF‐mutant collections (Davis et al., 2002; Homann, Dea, Noble, & Johnson, 2009). These TFs modulate the expression of target genes within highly intricate regulatory networks involving direct cross‐talks between TFs (Nobile et al., 2012) and the regulation of the expression of specific effectors such as adhesins (*ALS1* and *HWP1*; Nobile et al., 2012; Nobile & Mitchell, 2005), cell‐surface genes, and hyphal/virulence‐associated genes (Finkel et al., 2012). The biological circuitries under the control of TFs involved in the expression of C. albicans pathogenicity traits can be inferred from a combination of genome‐wide expression and location (ChIP‐chip/Seq) technologies (Chen, Pande, French, Tuch, & Noble, 2011; Perez et al., 2013; Znaidi, Nesseir, Chauvel, Rossignol, & d'Enfert, 2013; Znaidi et al., 2014). As an example, the role of the Sfu1p and Sef1p TFs in C. albicans ability to respectively act as a commensal and a systemic‐infection pathogen through modulation of iron homoeostasis has been elegantly shown by combining ChIP‐chip and transcriptomics experiments (Chen, Pande, et al., 2011). The same approaches also demonstrated that *RTG1*, *RTG3*, *TYE7*, and *LYS144* mediate GI tract colonisation by controlling the expression of genes involved in the acquisition and metabolism of specific nutrients, reflecting the importance of nutrient sensing/uptake during C. albicans commensalism (Perez & Johnson, 2014). Such systems biology‐driven strategies are therefore cornerstone for mapping biological networks operating during C. albicans interaction with the host.

Gene overexpression is another powerful genetic approach for the discovery of pathways and phenotypes (Chua et al., 2006; Douglas et al., 2012; Sopko et al., 2006). It mimics gain‐of‐function mutations, complements loss‐of‐function phenotypes, and allows the function of both essential and non‐essential genes to be investigated (Prelich, 2012). Because diploidy and lack of a complete sexual cycle hamper the use of classical genetics in C. albicans, gene overexpression is regarded as an attractive alternative strategy for performing functional large‐scale studies in this pathogen. We have previously established C. albicans strain collections for conditional gene‐overexpression using the pNIM1 (Park & Morschhauser, 2005) and pNIMX (Chauvel et al., 2012) systems that respectively enable moderate and potent tetracycline derivative‐induced expression (Chauvel et al., 2012). Strains from our collections carry unique 20‐bp signature tags that allow simultaneous phenotyping in mixed‐population experiments (Chauvel et al., 2012). We successfully used a collection with moderate, pNIM1‐driven, overexpression of 531 open‐reading frames (ORFs) for the identification of genes involved in biofilm formation, one of the major pathogenicity traits of C. albicans (Cabral et al., 2014). In the current report, we established a new library carrying 572 signature‐tagged strains with potent, pNIMX‐driven, conditional overexpression (Chauvel et al., 2012). We used it in a mouse model of GI tract colonisation to propose a new role for the TF Crz2p: the regulation of processes controlling the ability of C. albicans to efficiently proliferate within the host.

## 2 RESULTS

### 2.1 Construction of a new signature‐tagged overexpression strain collection with enhanced tetracycline promoter‐driven overexpression

**Figure 1:**
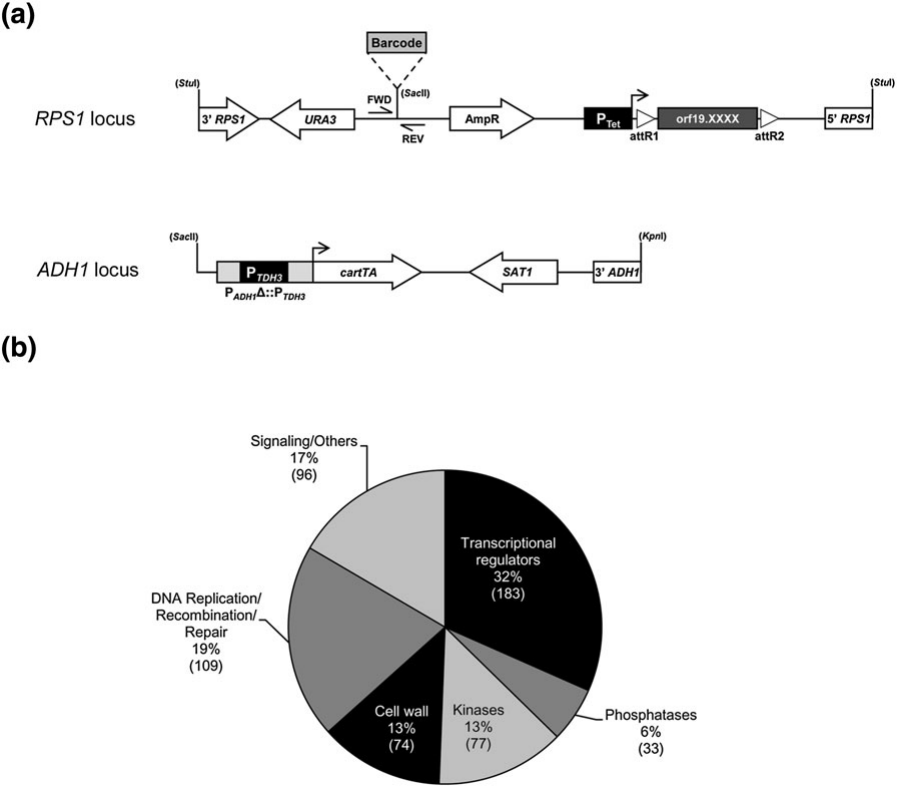
Construction of a new tetracycline‐inducible overexpression strain collection with enhanced overexpression efficiency. (a) Schematic representation of the *Stu*I‐linearised, signature‐tagged (Barcode), overexpression vector that was used to create the 572‐strain collection. Expression of each of the 572 open‐reading frames (ORFs; orf19.XXXX; dark grey rectangle) is under the control of the tetracycline‐inducible promoter (P_Tet_, black box), which is activated in the presence of doxycycline (horizontal arrow). The overexpression cassette is integrated at the *RPS1* locus following a *Stu*I digestion (3′ *RPS1*, 5′ *RPS1*). The ORF is flanked by the lambda‐phage attachment sequences R1 and R2 (open triangles) that allowed recombination‐mediated transfer of ORFs from an entry vector collection to the destination barcoded vectors. A unique 20‐bp sequence (Barcode) serves as a strain identifier and allows the relative abundance of each strain to be quantified in mixed‐population experiments. Every strain also carries the *Sac*II, *Kpn*I‐linearised pNIMX construct (Chauvel et al., 2012) integrated at the *ADH1* locus and carrying the Candida albicans reverse tetracycline transcriptional activator (*cartTA*) placed under the control of the *TDH3* promoter (P_*TDH3*_). Transformant selection markers are depicted with open arrows. (b) Pie chart showing the functional categories of the 572 ORFs included in the overexpression strain collection. The number of ORFs is indicated between parentheses

We previously generated a signature‐tagged C. albicans overexpression collection totalling 274 ORFs with tetracycline‐derivative inducible expression (P_*TET*_) based on the pNIMX expression system (Figure 1a; Chauvel et al., 2012). In this study, we expanded this collection to encompass 572 ORFs (~10% genome coverage), including genes encoding (or predicted to encode) transcriptional regulators (183 ORFs), phosphatases (33 ORFs), kinases (77 ORFs), cell wall genes (74 ORFs), DNA replication/recombination/repair genes (109 ORFs), and signalling/other category genes (96 genes; Table S1, Figure 1b).

### 2.2 Identification of genes whose overexpression alters both Candida albicans fitness and morphology in vitro

**Figure 2:**
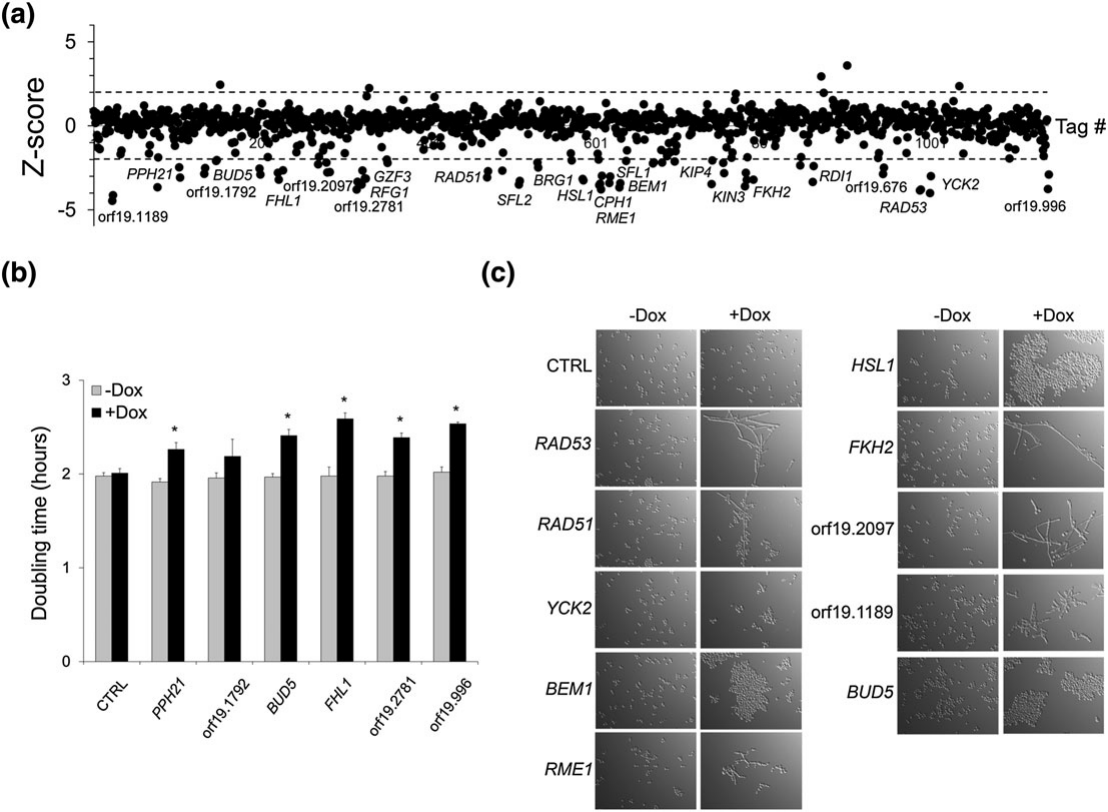
Competitive fitness profiling of Candida albicans overexpression strains under standard laboratory growth conditions. (a) The effect of gene overexpression on cell growth was tested in liquid YPD medium at 30°C under vigorous shaking (standard laboratory growth conditions, normoxia) in the absence or presence of 40 μg ml^−1^ doxycycline (Dox) for 18 generations. The experiment was performed using *n* = 3 biological replicates. Samples were subjected to genomic DNA extraction, polymerase chain reaction‐amplification, indirect fluorescent dye labelling (Dox‐treated sample: Cy5‐labelled; untreated control: Cy3‐labelled), and hybridization to a barcode microarray with both forward and reverse‐complemented probes for each tag (two black circles next to each gene name/orf19 nomenclature represent forward and reverse‐complemented probe sequences). Fitness scores (*Z*‐score for each tag) are shown on the *y* axis. The corresponding probe number ranked using the orf19 nomenclature in ascending order is shown on the *x* axis. *Z*‐score calculations were performed using ArrayPipe v2.0. Dashed lines correspond to the *Z*‐score values +2.0 (upper line) and −2.0 (lower line). Names or orf19 nomenclature of the genes whose overexpression alters strain fitness are shown. (b) Confirmation of the microarray data by liquid growth assay (YPD, 30°C) of strains overexpressing *PPH21*, orf19.1792, *BUD5*, *FHL1*, orf19.2781, and orf19.996, together with a control strain carrying the empty vector (CTRL) grown 3 times independently in a 96‐well plate using a TECAN Infinite M200 device (see Section 4). Doubling time in hours (average from *n* = 3 biological replicates and error bars denote standard deviations) is indicated on the *y* axis for each strain grown in the absence (grey bar, −Dox) or presence (black bar, +Dox) of 40 μg ml^−1^ Dox; statistical significance was assigned (*P* < 0.05, asterisks) by performing a two‐tailed Student's *t* tests. (c) Phenotypic analysis of a subset of strains overexpressing the indicated genes or control strain (CTRL). Strains were microscopically examined (40× magnification) immediately after being subjected to the fitness profiling assay described in (b) +Dox, dox treatment (40 μg ml^−1^); −Dox, untreated samples

We used our new collection to identify genes whose overexpression alters C. albicans fitness during growth under standard laboratory conditions (i.e., rich medium, normoxia at 30°C). The presence of a unique 20‐bp barcode sequence (Figure 1a) allows quantification of strain abundance during competitive growth, as we previously showed (Cabral et al., 2014). The 572 strains were competitively grown in Yeast Extract‐Peptone‐Dextrose (YPD) medium at 30°C for 20 generations in the absence or presence of 40 μg ml^−1^ doxycycline (Dox), followed by genomic DNA extraction, barcode polymerase chain reaction (PCR)‐amplification, cyanine‐dye labelling, and hybridization to barcode arrays (see Section 4 for details). We found 25 genes whose overexpression decreased C. albicans fitness (Figure 2a, Table S2, *Z*‐score ≤ −2; *P* < 0.05), including 10 genes involved (or predicted to be involved) in DNA damage response or cell‐cycle progression (*RAD53*, *RAD51*, *RME1*, orf19.2781, *KIN3*, *BEM1*, *HSL1*, orf19.2097, orf19.1792, and *PPH21*), 9 genes involved in hyphal development (*CPH1*, *SFL2*, *BRG1*, *SFL1*, *YCK2*, *RDI1*, *FKH2*, *KIP4*, and *RFG1*), and 6 genes encoding signalling proteins or exerting other/yet unknown functions (orf19.996, orf19.676, *GZF3*, *FHL1*, *BUD5*, and orf19.1189; Figure 2a). Our screen reconfirmed the genes that we previously showed to affect C. albicans fitness using the pNIM1 system (*RAD53*, *RAD51*, *SFL2*, and orf19.2781; Cabral et al., 2014) and identified additional hits (Figure 2a), suggesting that enhancing overexpression levels (pNIMX achieves approximately fivefold higher overexpression levels as compared with pNIM1) increases the sensitivity of our assay. We validated our microarray data using liquid growth assay of individually grown strains (Figure 2b, see Section 4). Interestingly, overexpression of some of the fitness‐defect genes altered cell morphology (*RAD53*, *RAD51*, *RME1*, *FKH2*, orf19.2097, and orf19.1189) or induced cell–cell aggregation (*YCK2*, *BEM1*, *HSL1*, and *BUD5*; Figure 2c). As morphology alterations affect turbidity measurements (Cabral et al., 2014), these strains were omitted from our liquid growth assay. Taken together, these data validated our competitive phenotypic profiling with the new pNIMX collection and identified new genes whose overexpression affects C. albicans fitness, morphology, and cell–cell aggregation.

### 2.3 Identification of genes whose overexpression alters colonisation of the mammalian GI tract

**Figure 3:**
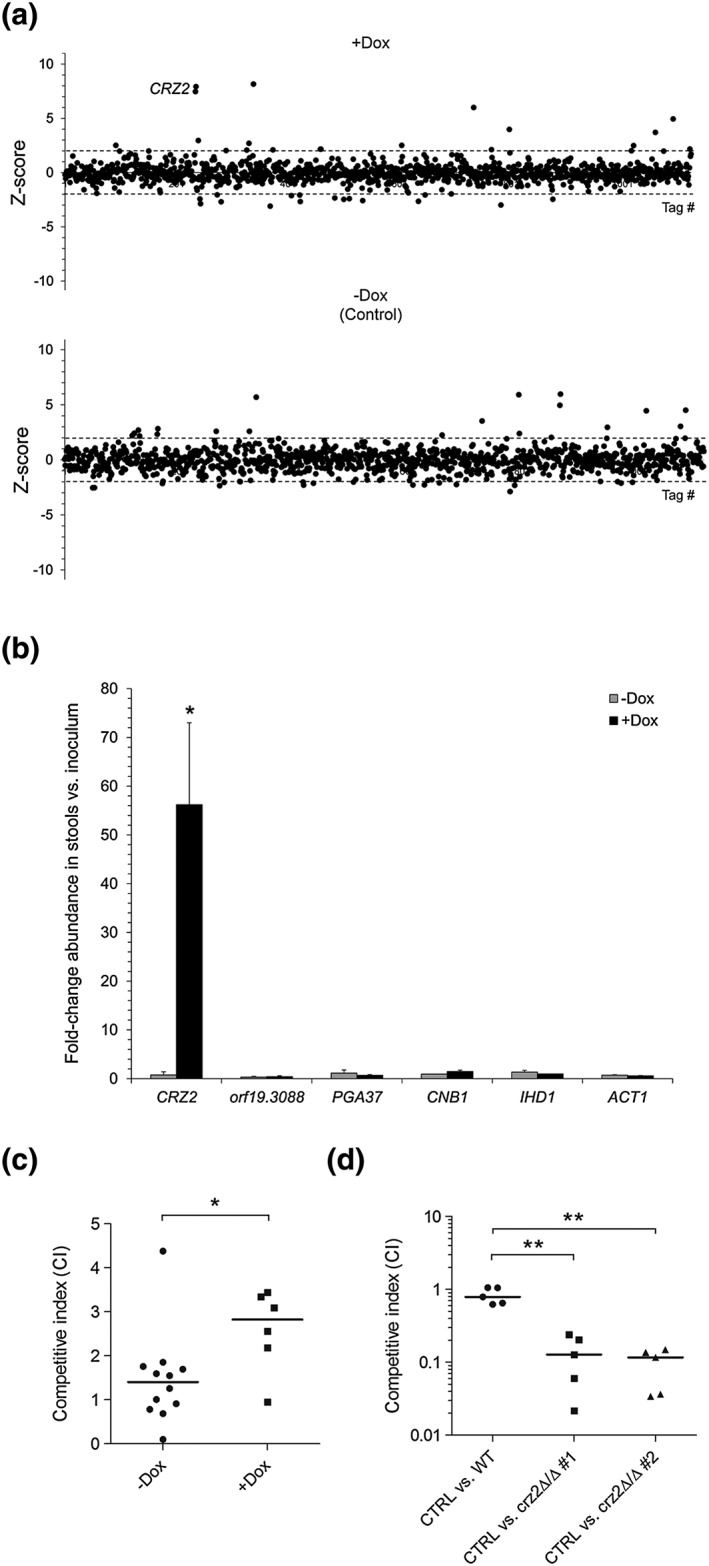
*CRZ2* contributes to Candida albicans fitness during gastrointestinal colonisation. (a) Genomic DNA was extracted from faeces of BALB/c female mice at Day 10 post‐gavage with an inoculum of 572 P_*TET*_‐inducible‐overexpression strains. Barcodes were polymerase chain reaction‐amplified from both faecal (Cy5‐labelled) and inoculum (Cy3‐labelled) genomic DNA and hybridized to barcode arrays (Figure S1). Data are presented as described in legend to Figure 2a. The *CRZ2* tag displays increased abundance (positive *Z*‐score) in dox‐treated mice (+Dox, *n* = 5 mice, *P* = 0.01) but not in dox‐untreated animals (−Dox, Control, *n* = 4 mice). (b) Pooled faecal samples, at Day 10 post‐gavage, from two independent cages housing three dox‐treated (+Dox, black bars) or three dox‐untreated (−Dox, grey bars) mice were subjected to genomic DNA extraction and up to seven quantitative polymerase chain reaction assays to determine the average fold‐change abundance (*y* axis, error bars denote standard deviations) of strain P_*TET*_‐*CRZ2* (*CRZ2*) as compared with its abundance in the inoculum. Strains orf19.3088, *PGA37*, *CNB1*, and *IHD1* were used as negative controls, and *ACT1* served as a normalisation control. One representative result out of two is shown. Statistical significance was assessed by a two‐tailed Student's *t* test (asterisk, *P* < 0.05). (c and d**)** Median competitive indexes (CIs, *y* axis) at Day 4 post‐gavage of strains P_*TET*_‐*CRZ2* (green fluorescent protein [GFP]‐labelled) versus control (blue fluorescent protein [BFP]‐labelled) in dox‐treated (+Dox, *n* = 6) and dox‐untreated (−Dox, *n* = 12) mice (c) and two independent *crz2*Δ/*crz2*Δ mutants (crz2Δ/Δ #1 and #2, GFP‐labelled) versus control (CTRL, BFP‐labelled) or the parental wild‐type strain (WT, GFP‐labelled) versus control (CTRL, BFP‐labelled) (d) were determined by flow cytometry analyses (see Section 4). Statistical significance was assessed using a non‐parametric Mann–Whitney test (two‐tailed; ^*^ *P* < 0.05; ^**^ *P* < 0.01). Dox: doxycycline

We screened our collection, in vivo, for genes whose overexpression alters C. albicans ability to colonise the murine gut (Figure S1 for a schematic representation of our strategy). Mice were given gentamycin‐ and streptomycin‐containing drinking water, supplemented (*n* = 5 mice) or not (control, *n* = 4 mice) with 2 mg ml^−1^ doxycycline, and then inoculated by gavage with ~5 × 10^7^ cells from the 572‐strain pool (Figure S1, see Section 4). Stools were collected 10 days post‐gavage. Total genomic DNA was extracted from both faeces and inoculum samples, and the relative abundance of the strains was assessed by microarrays (Figure S1). We found one hit out of the 572‐competing strains displaying increased abundance in dox‐treated mice (Figure 3a, upper panel, +Dox) and unaltered abundance in dox‐untreated animals (Figure 3a, lower panel, −Dox). This hit matched *CRZ2* that encodes a zinc finger TF of the Cys_2_His_2_ family that was previously shown to be required for adaptation to low pH (Kullas, Martin, & Davis, 2007). To validate our microarray data, we performed quantitative PCR (qPCR) assays and specifically quantified the relative abundance of the *CRZ2* overexpression strain in pooled mouse stools from dox‐treated/untreated cages (housing three mice each) as compared with its abundance in the inoculum (Figure 3b). The *CRZ2*‐overexpressing strain was more abundant in stools from dox‐treated mice, whereas its abundance was unaltered in stools from dox‐untreated animals (Figure 3b). Unchanged strain abundance was observed for four randomly selected strains (orf19.3088, *PGA37*, *CNB1*, and *IHD1*, Figure 3b). Under standard laboratory growth conditions, in vitro, the abundance (Figure 2, Table S2) or growth rate (Figure S2A) of the *CRZ2*‐overexpressing strain was unaltered, reflecting the specificity of our in vivo assay. We have also inspected colony size/morphology (e.g., GUT phenotype) and cellular morphology following passage through the mouse. We did not detect clear differences in morphology and/or size. We have also tested whether overexpression of *CRZ2* could alter hyphal growth in both rich and hyphae‐inducing media and found no difference compared with the control strain.

To test whether the *CRZ2* overexpression phenotype could be reproduced in a 1:1 competition assay, we gavaged dox‐treated and dox‐untreated mice with an inoculum containing an equal mixture of a strain co‐expressing the green fluorescent protein (GFP, under the control of P_*TDH3*_) and *CRZ2* (under the control of P_*TET*_) versus a strain expressing the blue fluorescent protein (BFP, under the control of P_*TDH3*_) and carrying an empty‐vector control (see Section 4). Stools were recovered 4 days post‐gavage, homogenised, and plated on gentamycin‐ and chloramphenicol‐containing YPD medium during 2 days at 30°C. The resulting C. albicans colony‐forming units (CFUs) were pooled, and the relative abundance of strains carrying P_*TET*_‐*CRZ2* (GFP) and control plasmid (BFP) was quantified with fluorescence‐activated cell sorting (Figure 3c, see Section 4). The CFUs from dox‐treated mice displayed increased median competitive index (CI: ~2.9) for the GFP‐positive (*CRZ2*) strain as compared with the BFP control strain (Figure 3c), whereas those from dox‐untreated animals showed a median CI close to 1.0 (Figure 3c). The effect of deleting *CRZ2* was also examined using the same strategy, except that dox treatment was omitted from the assay because we used non‐conditional *CRZ2* deletion mutants (see Section 4). We tested two independently generated *crz2*−/− strains and found that both mutants failed to maintain efficient colonisation, as judged by the significant decrease in their median CIs (Figure 3d).

We further measured the relative abundance of the *CRZ2* overexpression and deletion mutants at later time points using our 1:1 competition assay (Days 10 and 14). Surprisingly, the effect of *CRZ2* overexpression and deletion on GI colonisation was not sustained over time (Figure S2B,C), which may reflect an adaptive process confined to the upper sections of the digestive tract (see below). Yet, increased abundance of the *CRZ2*‐overexpressing strain was still detected in our 1:571 competitive screen on Day 10 post‐gavage (Figure 3b). To explain this discrepancy, we simulated competitive growth of a strain with a fitness of either 2.2 or 2.1 relative to a strain with a fitness of 2.0 using two different ratios: 1:1 and 1:571 (Table S5). We calculated the corresponding CIs for cultures with a 1:1 ratio versus those with a 1:571 ratio (Table S5). Consistent with our observation, we found that CIs evolve differently according to the competition ratio and time, inuring to the benefit of the 1:571 comparison (see Graph in Table S5).

### 2.4 Crz2p activates the hypoxic transcriptional program and modulates the expression of cell‐surface genes

**Figure 4:**
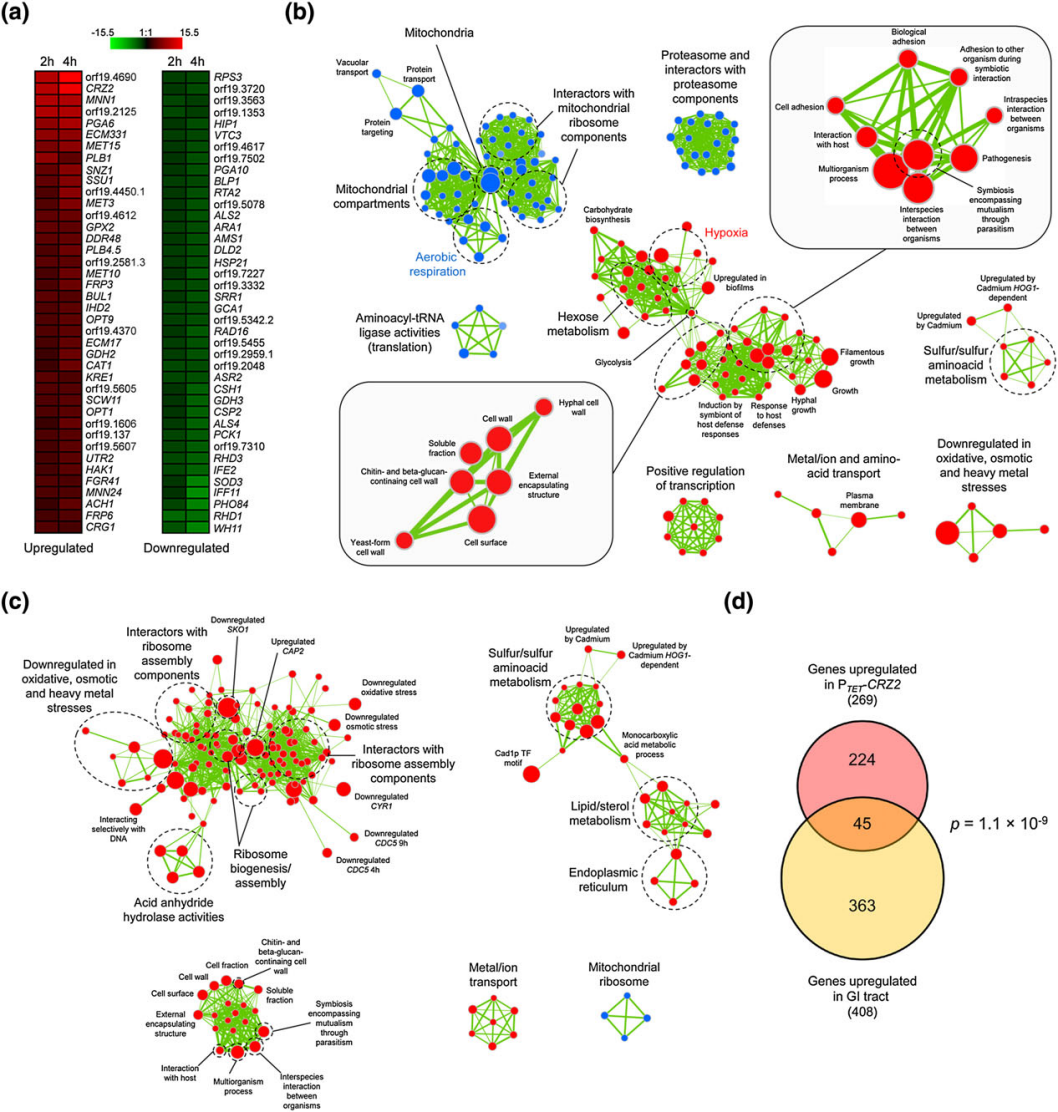
Transcriptomic analysis of *CRZ2* overexpression strains. (a) Heat maps of the 40 highest transcriptionally modulated genes (absolute n‐fold changes are shown) in P_*TET*_‐*CRZ2* transcript profiling data at time points 2 and 4 hr post‐induction with 40 μg ml^−1^ doxycycline (combination of three biological replicates in each condition). The most upregulated (descending signal intensity, sorted by average expression between 2 and 4 hr, left panel) or downregulated (ascending signal intensity, sorted by average expression between 2 and 4 hr, right panel) genes in dox‐treated versus untreated cells are indicated with their corresponding name or orf19 nomenclature on the right side of each panel. Heat maps were constructed using Genesis version 1.7.6 (Sturn, Quackenbush, & Trajanoski, 2002). (b and c) Gene‐set enrichment analyses maps at time points 2 hr (b) and 4 hr (c) post‐induction of *CRZ2* gene expression. Functional enrichment among the upregulated and downregulated genes is depicted with red and blue spheres, respectively. Sphere size is proportional to the number of genes. Network motif thickness is proportional to the extent of overlap in the list of genes between each node (blue/red spheres). Selected functional categories are indicated with dashed circles. Highlighted subnetworks are zoomed in (open large boxes in [b]). (d) Venn diagram of the overlap (45 genes) between genes that are induced by P_*TET*_‐driven overexpression of *CRZ2* (269 genes) and those that are transcriptionally upregulated in mouse gastrointestinal (GI) tract (408 genes) as identified by Rosenbach et al. (2010). Statistical significance (*P* = 1.1 × 10^−9^) was assessed using a hypergeometric test. TF: transcription factor

Crz2p was shown to regulate the expression of a subset of Zap1p targets involved in biofilm formation using nanoString transcript profiling of 293 genes (Finkel et al., 2012). To comprehensively define the regulatory network of Crz2p and better understand how *CRZ2* contributes to colonisation of the GI tract, we performed whole‐genome transcript profiling of the strain overexpressing *CRZ2*. The P_*TET*_‐*CRZ2* strain was grown in YPD medium at 30°C, in the absence or presence of dox, for 2 and 4 hr to investigate early (2 hr, those that could reflect direct targets) versus later (4 hr, including direct and indirect targets) transcriptional programming (see Section 4). At time point 2 hr, 110 and 66 genes were respectively upregulated (fold‐change ≥1.5, *P* < 0.05) and downregulated (fold‐change ≤−1.5, *P* < 0.05) in response to *CRZ2* induction (Figure 4a, Table S6). At time point 4 hr, 220 and 205 genes were respectively upregulated and downregulated using the same criteria (Figure 4a, Table S6). Among the P_*TET*_‐*CRZ2* upregulated genes, we found a high proportion of those encoding (or predicted to encode) cell‐surface proteins (e.g., *PGA6*, *ECM331*, *PLB1*, *PLB4.5*, *KRE1*, and *SCW11*), mannosyltransferases (e.g., *MNN1*, *MNN24*, *MNN22*, and *BMT5*), proteins involved in methionine/cysteine metabolism (e.g., *MET15*, *MET3*, *MET10*, and *ECM17*), and small molecule/amino‐acid transporters (e.g., orf19.4690, *SSU1*, *FRP3*, *FRP6*, and *OPT1*; Figure 4a, Table S6). On the other hand, many cell wall/surface genes (e.g., *IFF11*, *RHD3*, *ALS4*, *CSP2*, *CSH1*, *ALS2*, and *PGA10*) and genes involved in signalling (*ASR2* and *SRR1*) and carbohydrate metabolism (*PCK1*, *DLD2*, and *ARA1*) were downregulated (Figure 4a, Table S6). As a control, dox treatment alone does not significantly alter gene expression (Figure S3). Only 13 and 9 genes were upregulated and downregulated with maximum fold‐change values of 2.0 and −2.5, respectively (Table S7), including 10 genes displaying increased fold‐change due to a skew in the distribution of high‐intensity signal (Figure S3B and Table S7).

To get a global view of the metabolic processes and pathways that were significantly transcriptionally modulated by *CRZ2* induction, we performed gene‐set enrichment analyses (GSEA) and used the Cytoscape GSEA plugin (Sellam et al., 2014) to map the biological pathways and gene sets that are enriched among the upregulated (red spheres) and downregulated (blue spheres) genes at time points 2 hr (Figure 4b) and 4 hr (Figure 4c; see Section 4). During the early (2 hr) transcriptional response to *CRZ2* induction, there was a striking enrichment of processes pertaining to mitochondria and proteasome function among the downregulated genes (Figure 4b, blue sphere‐containing networks), with a noticeable downregulation of aerobic respiration (Figure 4b, marked in blue). Conversely, hypoxic genes were significantly enriched among the upregulated genes (Figure 4b, marked in red), together with processes pertaining to hexose metabolism (e.g., glycolysis and carbohydrate biosynthesis), sulfur amino‐acid metabolism, positive regulation of transcription, and metal/small molecule transport (Figure 4b, red sphere‐containing networks). The genes encoding cell wall components (Figure 4b, lower boxed network) and those participating in interaction with the host (Figure 4b, upper boxed network) were also remarkably enriched. At time point 4 hr, ribosome biogenesis and assembly along with gene sets that are downregulated during various stresses (e.g., oxidative, osmotic, and heavy metal stresses) or by specific signalling proteins (Cyr1p, Cdc5p, Cap2p, and Sko1p) were significantly overrepresented among the *CRZ2*‐induced genes (Figure 4c, upper left network). The sulfur amino‐acid metabolism genes and those encoding cell wall components or participating in C. albicans–host interaction were still enriched (Figure 4c, upper right and lower left networks), whereas genes involved in lipid/sterol metabolism and those encoding components (or functional components) of the endoplasmic reticulum (ER) became overrepresented (Figure 4c, upper right subnetworks).

Rosenbach, Dignard, Pierce, Whiteway, and Kumamoto (2010) analysed the genome‐wide transcriptional changes of C. albicans during GI tract colonisation as compared with laboratory growth conditions. They identified a set of 408 genes specifically upregulated in the mouse gut. We compared the list of genes that are significantly upregulated by P_*TET*_‐*CRZ2* to those identified in Rosenbach et al. (Figure 4d). Among the 408 genes that are induced in the gut, 45 were also upregulated by P_*TET*_‐*CRZ2* at time points 2 or 4 hr (out of 269 genes, Figure 4d), yielding a ~2.6‐fold enrichment of mouse gut‐induced genes in our dataset (*P* = 1.1 × 10^−9^ using a hypergeometric test, Figure 4d). Interestingly, the GO terms “glycolytic process” (*ADH1*, *ENO1*, *FBA1*, and *TDH3*; *P* = 1.11 × 10^−3^), “cell surface” (*ADH1*, *ENO1*, *FBA1*, *HWP1*, *IHD1*, *PGA54*, *SAP9*, *SUN41*, *TDH3*, and *TOS1*; *P* = 5.25 × 10^−5^), and “symbiosis, encompassing mutualism through parasitism” (*ADH1*, *ENO1*, *FBA1*, *HWP1*, *SAP9*, *SUN41*, and *TDH3*; *P* = 6.68 × 10^−3^) were particularly overrepresented among the 45 gut‐ and *CRZ2*‐induced genes, possibly reflecting a role of *CRZ2* in adaptation to hypoxia and the modulation of cell‐wall function during GI colonisation. We validated our expression microarray data by quantitative reverse transcription‐PCR (qRT‐PCR) analyses of selected genes using *ACT1* as a negative control for differential expression (Figure S4).

Taken together, our results indicate that *CRZ2* overexpression turns off aerobic respiration and activates the hypoxic transcriptional signatures, while inducing the expression of cell wall genes together with genes involved in GI colonisation and interaction with the host.

### 2.5 Crz2p binds to the promoter of mannosyltransferase‐ and cell wall protein‐encoding genes

**Figure 5:**
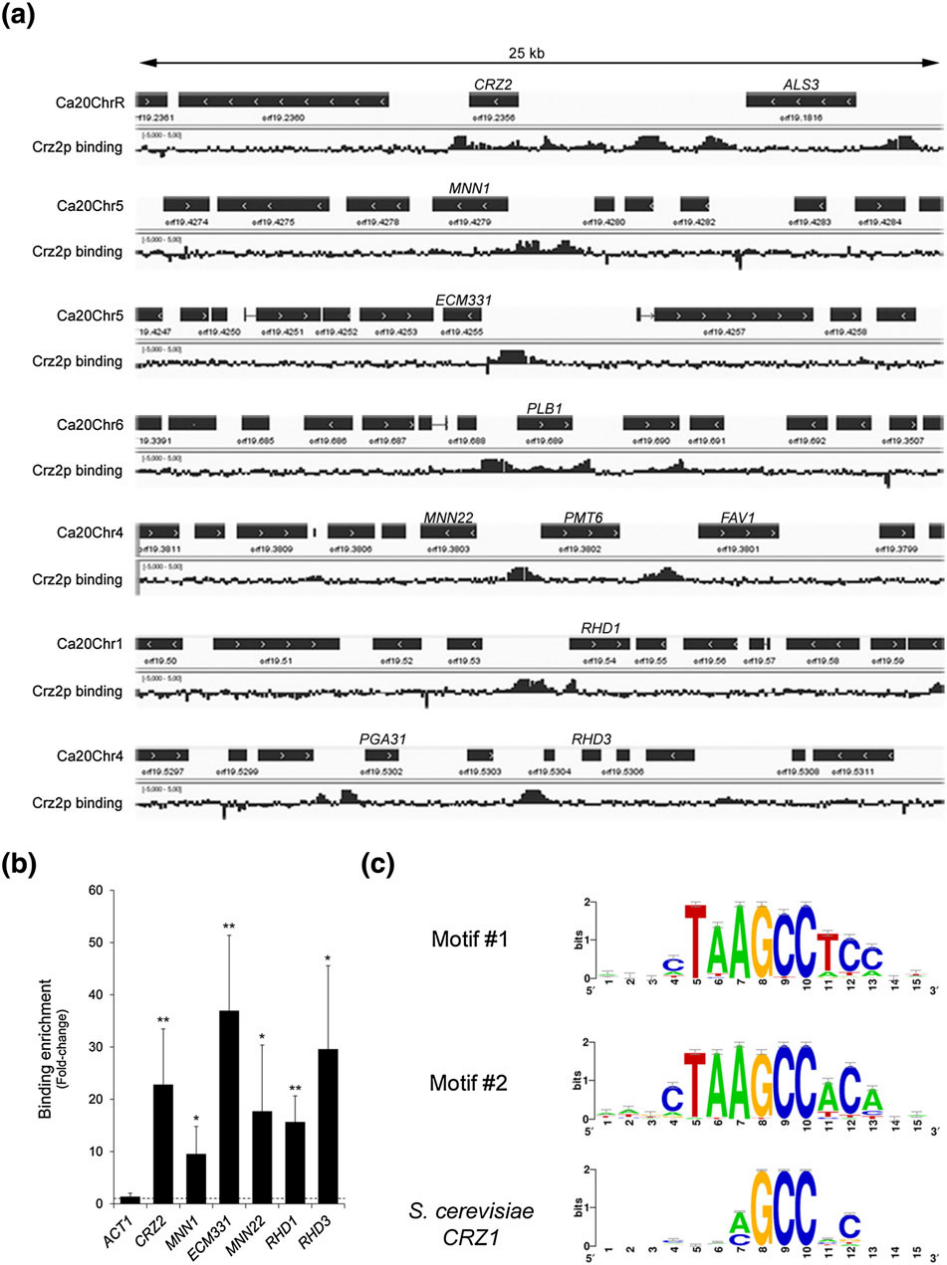
Genome‐wide location of transcription factor Crz2p. (a) Crz2p occupancies along 25‐kb intervals of selected locations from the Candida albicans genome (Assembly 20, the corresponding chromosome numbers are indicated at the left of each panel). Plotted are the relative signal intensities of the 60‐bp probes covering the whole C. albicans genome following enrichment of the tandem affinity purification (TAP)‐tagged Crz2p‐coimmunoprecipitated DNA relative to DNA from a mock immunoprecipitation (i.e., in an untagged‐strain background). Data from one ChIP‐chip experiment out of two are shown. Some binding‐enrichment signals extend beyond the maximum graduations (−5.0 and +5.0 fold‐enrichment). The orientation of each open‐reading frames is depicted by the arrowed black rectangle. Binding maps were generated using the Integrated Genomics Viewer genome browser (Thorvaldsdottir et al., 2012). (b) Quantification of DNA enrichment following immunoprecipitation of TAP‐tagged Crz2p at the promoters of *CRZ2*, *MNN1*, *ECM331*, *MNN22*, *RHD1*, and *RHD3* by quantitative polymerase chain reaction assays in strains C251, C252 (tagged), C255 and C257 (control strains; untagged). Bars represent absolute relative enrichment values (n‐fold, *y* axis) of TAP‐Crz2p coimmunoprecipitated DNA as compared with DNA from mock immunoprecipitation. Error bars denote standard deviations from the mean (using data from three quantitative polymerase chain reaction assays with two biological replicates in each assay, assumed as *n* = 6). All enrichment values were statistically significant using a two‐tailed Student's *t* test that compares binding enrichment values at the indicated loci to those at the *ACT1* locus (negative control; ^*^ *P* < 0.05; ^**^ *P* < 0.01). (c) Motif logos of conserved sequences in Crz2p‐enriched DNA fragments. DNA sequences encompassing ±250 bp around peak summits in Crz2p binding data were used as input for motif discovery by Regulatory Sequence Analysis Tool peak‐motifs (http://rsat.ulb.ac.be/rsat [Thomas‐Chollier et al., 2012]) algorithm. Two related high‐scoring motifs are shown (Motif #1 and Motif #2), together with the aligned Saccharomyces cerevisiae Crz1p motif (bottom logo)

To determine if the *CRZ2* transcriptionally modulated genes were directly regulated by Crz2p, we performed chromatin immunoprecipitation coupled with hybridization to whole‐genome tiling microarrays in strains expressing a tandem affinity purification (TAP)‐tagged versus a wild‐type version of *CRZ2*, both placed under the control of P_*TET*_ and grown for 4 hr in the presence of dox (see Section 4). Using the CisGenome peak finding algorithm (Ji et al., 2008), we identified 331 Crz2p‐binding peaks (Tables S8–S10). Among these peaks, 194 were located in promoter regions that were clearly associated with unique targets, 113 were located in promoters shared by two ORFs in opposite orientations, and the remaining 24 were found within ORF regions (Table S10). In many occurrences, more than one peak was found in the promoter of a given gene (Figure 5a, upper panel, *CRZ2* locus), suggesting the presence of more than one binding site for Crz2p or the occurrence of functional interactions with additional DNA‐binding proteins as previously observed (Znaidi et al., 2013). Overall, the 331 peaks matched 342 target genes, assuming that two ORFs sharing the same bound promoter are both direct targets of Crz2p. Many of the Crz2p‐bound targets encode cell wall proteins (e.g., *ALS3*, *ECM331*, *PLB1*, and *RHD3*) or mannosyltransferases (e.g., *MNN1*, *MNN22*, and *RHD1*; Figure 5a) and overlapped with genes transcriptionally modulated by Crz2p (Figure 4a). Crz2p also binds to its own promoter, suggesting a transcriptional autoregulatory loop (Figure 5a). We confirmed our ChIP‐chip data by qPCR analyses of selected targets (*CRZ2*, *MNN1*, *ECM331*, *MNN22*, *RHD1*, and *RHD3*) using *ACT1* as a negative control for Crz2p binding (Figure 5b).

We conducted motif‐enrichment analyses using DNA sequences encompassing ±250 bp around peak summits in Crz2p binding data. The Regulatory Sequence Analysis Tools peak‐motifs algorithm (http://rsat.ulb.ac.be/rsat [Thomas‐Chollier et al., 2012]) was used for motif discovery and motif comparison with known TF binding sites (see Section 4). We found two related high‐scoring motifs, 5′‐cTAAGCCtcc‐3′ and 5′‐cTAAGCCaca‐3′ (Figure 5c), with significance coefficients of >60 (*sig* scores = −log_10_ [E‐value], resulting from a binomial test). Motif comparison with known TF binding sites indicated that both motifs were similar to the Saccharomyces cerevisiae Crz1p motif (5′‐aGCCNC‐3′; Figure 5c, bottom panel), which may reflect the phylogenetic proximity of Crz1p and Crz2p.

### 2.6 The Crz2p regulatory network

**Figure 6:**
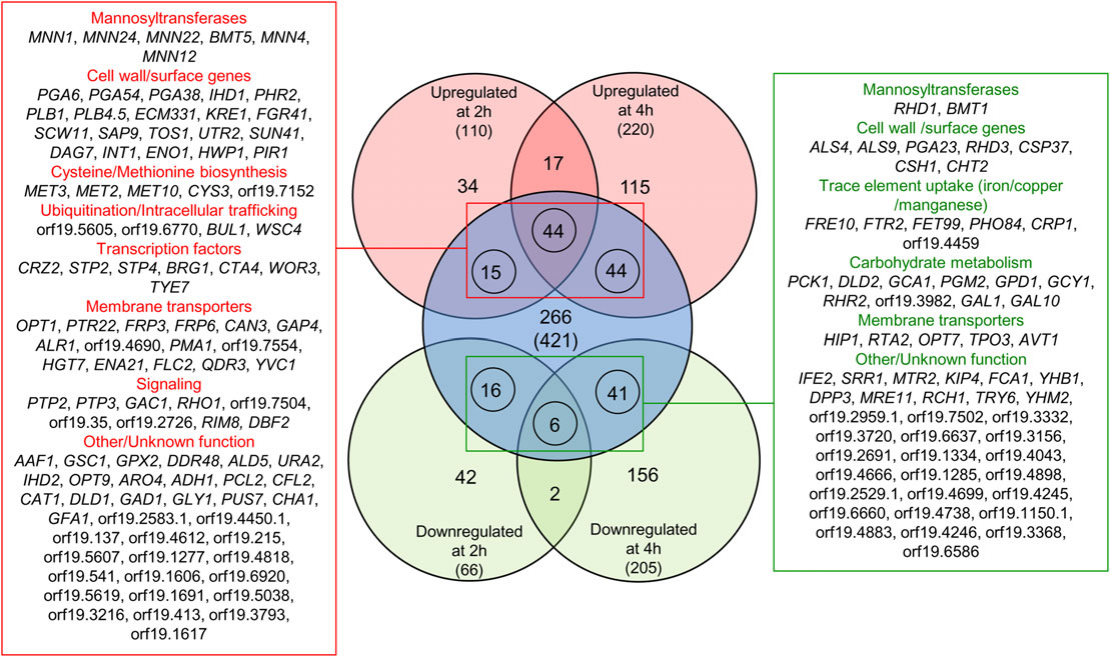
The Crz2p regulatory network. Venn diagrams of the overlap between genes that are transcriptionally modulated by P_*TET*_‐*CRZ2* at time points 2 and 4 hr (gene expression fold‐change ≥1.5; *P* < 0.05) and bound by Crz2p. Numbers in Venn diagrams indicate the number of genes, and those between parentheses indicate the total number of upregulated (light red circles), downregulated (light green circles), and bound (light blue circle) genes. Circled numbers indicate the number of genes that are both bound and transcriptionally modulated by Crz2p. The name of these genes (or their orf19 nomenclature) and the functional categories to which they belong are shown in the linked red (103 bound and upregulated genes) and green (63 bound and downregulated genes) boxes

We combined our genome‐wide expression and location data to map the direct regulatory interactions occurring between Crz2p and its target genes (Figure 6). This enabled the identification of additional bona fide Crz2p binding peaks matching 79 targets that were not detected by the peak‐finding algorithm, and these were added to the list of Crz2p targets (Table S9, Figure 6). We found 102 targets that were both bound and transcriptionally induced by Crz2p (Figure 6, red box) versus 63 genes that were both bound and transcriptionally repressed (Figure 6, green box), indicating that Crz2p acts as both activator and repressor of gene expression. A high proportion of the Crz2p directly induced genes encodes mannosyltransferases (GO:0005975, “Carbohydrate metabolic process,” *P* = 2.31 × 10^−3^), cell wall proteins (GO:0005618, “Cell wall,” *P* = 2.23 × 10^−7^), and membrane transporters (GO:0005215, “Transporter activity,” *P* = 0.044; Figure 6, red box). Similarly, a subset of genes encoding cell wall proteins, membrane transporters, and mannosyltransferases were directly downregulated (Figure 6, green box). Most of the membrane‐transporter genes are involved (or are predicted to be involved) in amino‐acid/peptide (*OPT1*, *OPT7*, *PTR22*, *CAN3*, *GAP4*, and *HIP1*) or metal/ion (*ALR1*, orf19.4690, *FLC2*, *FTR2*, *PHO84*, and *CRP1*) transport.

Taken together, our data indicate that Crz2p acts as both activator and repressor of gene expression and directly regulates the expression of genes potentially linked to cell wall function and to carbohydrate metabolism.

### 2.7 *CRZ2* mutants exhibit altered respiration

**Figure 7:**
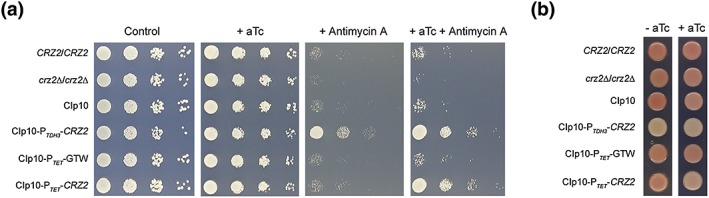
*CRZ2* mutants exhibit altered respiration. (a) Antimycin A susceptibility of *CRZ2*‐deficient (*crz2*Δ/*crz2*Δ) and *CRZ2*‐overexpressing (CIp10‐P_*TDH3*_‐*CRZ2* and CIp10‐P_*TET*_‐*CRZ2*) strains was tested by spot assay on SD plates supplemented with 20 μg ml^−1^ antimycin A or with 20 μg ml^−1^ antimycin A + 3 μg ml^−1^ anhydrotetracycline (aTc). The CIp10‐carrying (CIp10 and CIp10‐P_*TET*_‐GTW) and *CRZ2*/*CRZ2* strains were respectively used as a control. Plates were incubated at 30°C for 2 days. (b) Tetrazolium salt‐ (2,3,5‐triphenyltetrazolium chloride) containing overlay was poured on patches of the same strains to reconfirm respiration deficiency (final 0.05% triphenyltetrazolium chloride)

Our GSEA revealed that *CRZ2* overexpression turns on and off the hypoxic and cellular respiration transcriptional signatures, respectively (Figure 4b). We challenged growth of the *CRZ2* overexpression strain with Antimycin A, as C. albicans strains with reduced respiratory activity should show reduced sensitivity to this respiratory chain complex III inhibitor (Desai, van Wijlick, Kurtz, Juchimiuk, & Ernst, 2015; Figure 7a). Strikingly, a P_*TDH3*_‐*CRZ2* strain overexpressing *CRZ2* under the control of the constitutive P_*TDH3*_ promoter (Table S11) displayed marked resistance to Antimycin A (Figure 7a, + Antimycin A panel, compare CIp10 to CIp10‐P_*TDH3*_‐*CRZ2*). This phenotype was reproduced with the P_*TET*_‐*CRZ2* strain following addition of anhydrotetracycline (aTc) to the medium (Figure 7a, + aTc + Antimycin A panel, compare CIp10‐P_*TET*_‐GTW to CIp10‐P_*TET*_‐*CRZ2*). In contrast, loss of *CRZ2* resulted in increased sensitivity to Antimycin A (Figure 7a, compare *CRZ2/CRZ2* and *crz2*∆/*crz2*∆). We further confirmed the respiration deficiency phenotype of the *CRZ2*‐overexpressing strains using the triphenyltetrazolium chloride (TTC) overlay assay (Figure 7b), whereby reduction of TTC by the electron transport chain leads to the formation of a red product that accumulates within cells (Rich, Mischis, Purton, & Wiskich, 2001). As expected, the P_*TDH3*_‐*CRZ2* strain lacked the characteristic red/pink colour indicative of efficient respiratory activity in both aTc‐free and aTc‐supplemented media (Figure 7b, compare CIp10 to CIp10‐P_*TDH3*_‐*CRZ2*), whereas the P_*TET*_‐*CRZ2* strain displays respiratory deficiency only in the presence of aTc (Figure 7b, compare CIp10‐P_*TET*_‐GTW to CIp10‐P_*TET*_‐*CRZ2*). Thus, *CRZ2* expression appears to control respiratory activity in C. albicans.

### 2.8 *CRZ2* deletion alters the expression of Crz2p targets in response to hypoxia at 37°C

**Figure 8:**
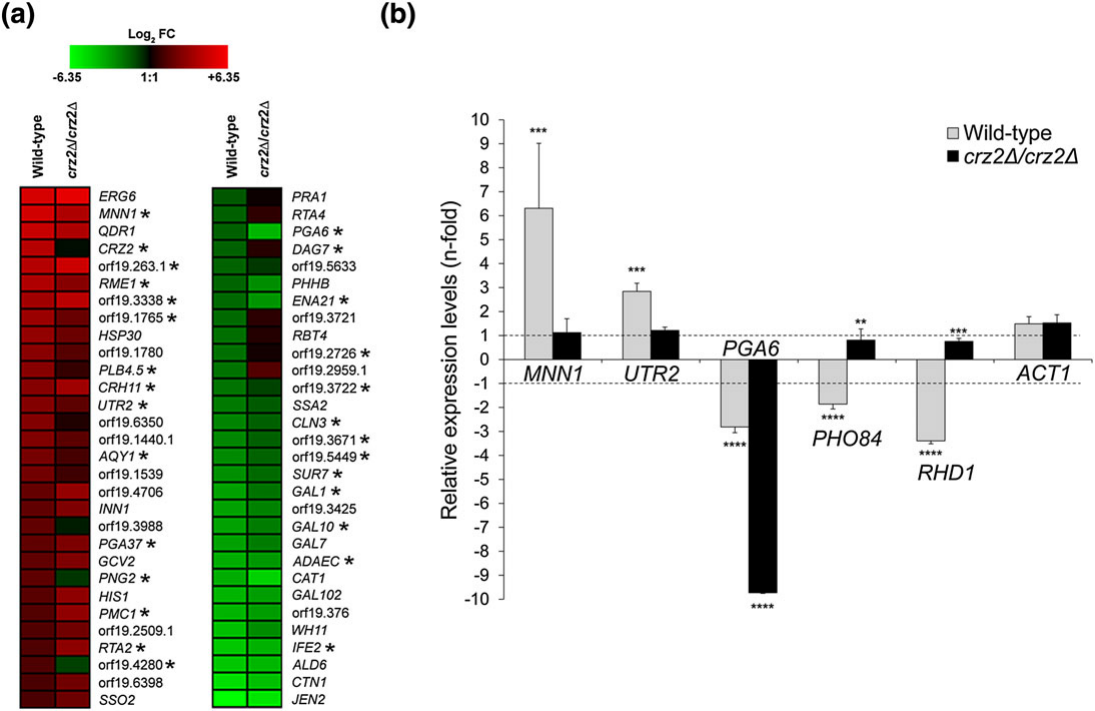
*CRZ2* deletion alters the expression of Crz2p targets in response to hypoxia at 37°C. (a) Heat maps of the 30 most upregulated (descending signal intensity, left panel) or downregulated (ascending signal intensity, right panel) genes in a *CRZ2*/*CRZ2* wild‐type strain (Wild‐type) following a shift from normoxia at 30°C to hypoxia at 37°C and their corresponding fold‐change intensities in the *CRZ2* homozygous mutant derivative (*crz2*Δ/*crz2*Δ) subjected to the same environmental perturbation (normoxia 30°C to hypoxia 37°C). All displayed genes show significant differential expression in the wild‐type strain as compared with the *crz2*Δ/*crz2*Δ mutant by analysis of variance analysis (*P* < 0.05). Genes are indicated with their corresponding name (or orf19 nomenclature) on the right side of each panel. Asterisks (*) indicate genes whose promoter was bound by Crz2p in ChIP‐on‐chip assay (Table S9). Heat maps were constructed using Genesis version 1.7.6 (Sturn et al., 2002). (b) The expression of *MNN1*, *UTR2*, *PGA6*, *PHO84*, *RHD1*, and *ACT1* (control) during growth under hypoxia at 37°C relative to their expression under normoxia at 30°C in the wild‐type strain (Wild‐type, light grey bars) versus the *crz2*Δ/*crz2*Δ mutant (*crz2*Δ/*crz2*Δ, black bars) was quantified by quantitative reverse transcription polymerase chain reaction. Bars represent the average relative change in RNA abundance of the indicated genes, and error bars denote standard deviations (*n* = 3 independently grown strains in each condition). Asterisks indicate significantly different gene expression levels as compared with the corresponding *ACT1* control using a standard Student's *t* test (^*^ *P* < 0.05; ^**^ *P* < 0.01; ^***^ *P* < 0.001; ^****^ *P* < 0.0001)

Based on the altered respiration of the *CRZ2* overexpression and deletion strains and the impact of *CRZ2* overexpression on the induction of the hypoxic program, we hypothesised that *CRZ2* could contribute to regulating adaptation to hypoxia when C. albicans is exposed to the GI tract environment. We analysed the transcriptome of a wild‐type strain relative to that of a *crz2*Δ/*crz2*Δ mutant, both grown under normoxia 30°C or under hypoxia 37°C (Figure 8a, Table S6, see Section 4, 37°C being combined with hypoxia to mimic the GI tract environment). We found that the expression levels of *CRZ2* itself were ~10‐fold induced (Figure 8a, Table S6), correlating with those reached using P_*TET*_‐driven overexpression (approximately eightfold induction, Figure 4a and Table S6). Importantly, the expression of many Crz2p bound targets was altered in the *crz2*Δ/*crz2*Δ mutant as compared with the wild‐type strain (Figure 8a, asterisks). The *crz2*Δ/*crz2*Δ mutant failed to fully activate or maintain the expression of a subset of P_*TET*_‐*CRZ2*‐upregulated genes, such as *MNN1*, *RME1*, *PLB4.5*, *UTR2*, *PGA6*, orf19.6350, and orf19.3988 (Figure 8a and Table S6). Similarly, a subset of the P_*TET*_‐*CRZ2* downregulated genes displayed altered expression in the *crz2*Δ/*crz2*Δ mutant, including *WH11*, *IFE2*, *ALD6*, *GAL10*, *PHO84*, *RHD1*, orf19.3721, orf19.2959.1, and orf19.3722 (Figure 8a and Table S6). We performed a qRT‐PCR assay to confirm our observations using primers that specifically amplify reverse‐transcribed RNA from *MNN1*, *UTR2*, *PGA6* (upregulated in P_*TET*_‐*CRZ2*), *PHO84*, *RHD1* (downregulated in P_*TET*_‐*CRZ2*), and *ACT1* (control, Figure 8b). Taken together, our data indicate that an oxygen‐scarce environment at 37°C induces transcriptional regulation by Crz2p.

### 2.9 Genetic perturbation of *CRZ2* under hypoxia 37°C alters Candida albicans sensitivity to highly acidic pH and bile salts

**Figure 9:**
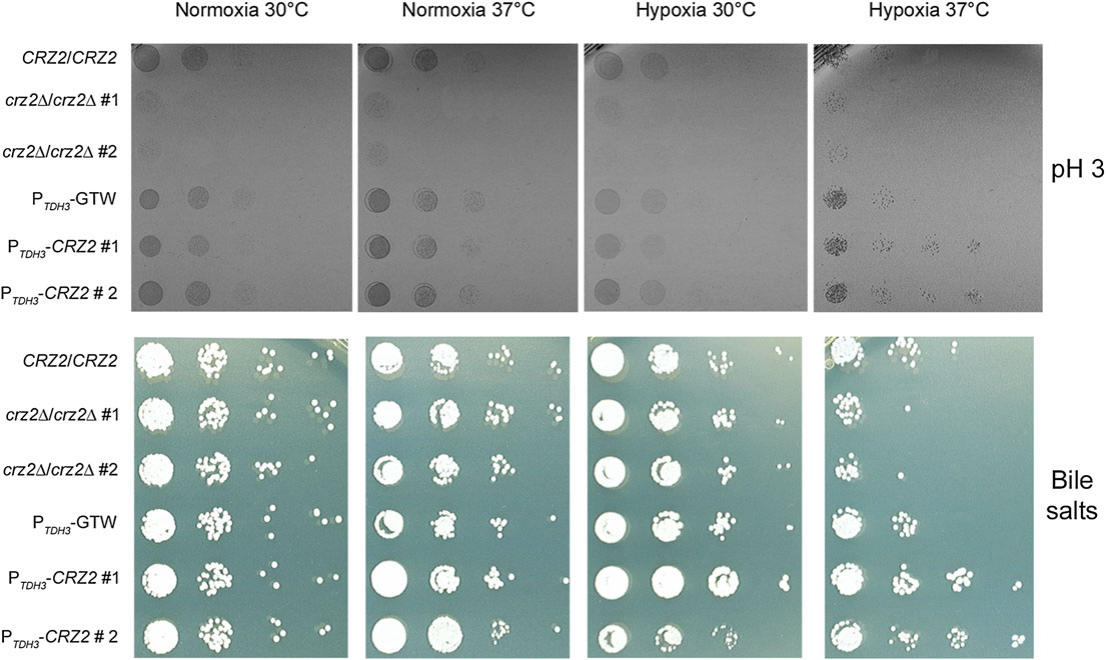
*CRZ2* is required for adaptation to acidic pH and bile salts under hypoxia at 37°C. pH 3‐ (upper panels) and bile salt‐ (lower panels) susceptibility phenotypes of two independent *crz2*Δ/*crz2*Δ (#1 and #2) and the corresponding wild‐type control (*CRZ2*/*CRZ2*) strains were analysed together with two independent P_*TDH3*_‐driven *CRZ2* overexpressers and the matched control strain (P_*TDH3*_‐GTW) by spot assay on SD plates supplemented with 150 mM HEPES at pH 3 and 0.1% bile salts, respectively. Plates were incubated under normoxia at 30°C or at 37°C and during hypoxia at 30°C or at 37°C for 2 and 7 days, respectively

Candida albicans adapts to different niches of the GI tract by optimising its growth and metabolism according to various parameters, including oxygen availability, pH variation (acidic in upper GI tract), bile‐salt, and (micro‐) nutrient availability (Noble, 2013; Perez & Johnson, 2014; Prieto, Correia, Pla, & Roman, 2016). Our observation that *CRZ2* is required for early colonisation of the GI tract could reflect an adaptive response of C. albicans to the upper section of the GI tract, including the stomach and proximal intestine, where pH is highly acidic and bile salts are abundant, respectively (Begley, Gahan, & Hill, 2005). We tested whether genetic perturbation of *CRZ2* alters C. albicans susceptibility to highly acidic pH (pH 3, Figure 9). We combined different temperature and oxygen‐availability parameters to further examine the specificity of the *CRZ2* phenotypes to hypoxia 37°C. As previously observed by Kullas et al. (2007), two independent *crz2*Δ*/crz2*Δ mutants already displayed sensitivity to acidic pH (Figure 9, upper panel). This phenotype was independent of growth temperature and oxygen availability (Figure 9, upper panel). Strikingly, strains overexpressing *CRZ2* showed marked resistance to acidic pH only under hypoxia 37°C (Figure 9, upper right panel, hypoxia 37°C), reinforcing the notion that *CRZ2* exerts its optimal activity under this condition.

We similarly tested the susceptibility of *CRZ2* overexpression and deletion strains on bile‐salt containing medium (Figure 9, lower panel). Growth of all tested strains was unaltered under normoxia 30°C, normoxia 37°C, and hypoxia 30°C (Figure 9, lower left and middle panels). Importantly, under hypoxia 37°C, the *CRZ2* deletion strains displayed increased sensitivity to bile salts, whereas the *CRZ2* overexpression strains were markedly resistant to the same compound (Figure 9, lower right panel, hypoxia 37°C). Both pH 3 and bile‐salt phenotypes were unlikely to reflect a more general *CRZ2*‐dependent stress sensitivity occurring under hypoxia 37°C, as growth of the corresponding strains on media containing cadmium (ER stress inducer) or copper (oxidative stress inducer) was not altered (Figure S5).

Taken together, our results further reinforce the requirement of hypoxia 37°C for Crz2p to optimally exert its function and suggest an important role of *CRZ2* in C. albicans adaptation to stresses encountered in the upper sections of the GI tract.

### 2.10 *CRZ2* overexpression confers sensitivity to the *N*‐glycosylation inhibitor tunicamycin and alters phosphomannan abundance

**Figure 10:**
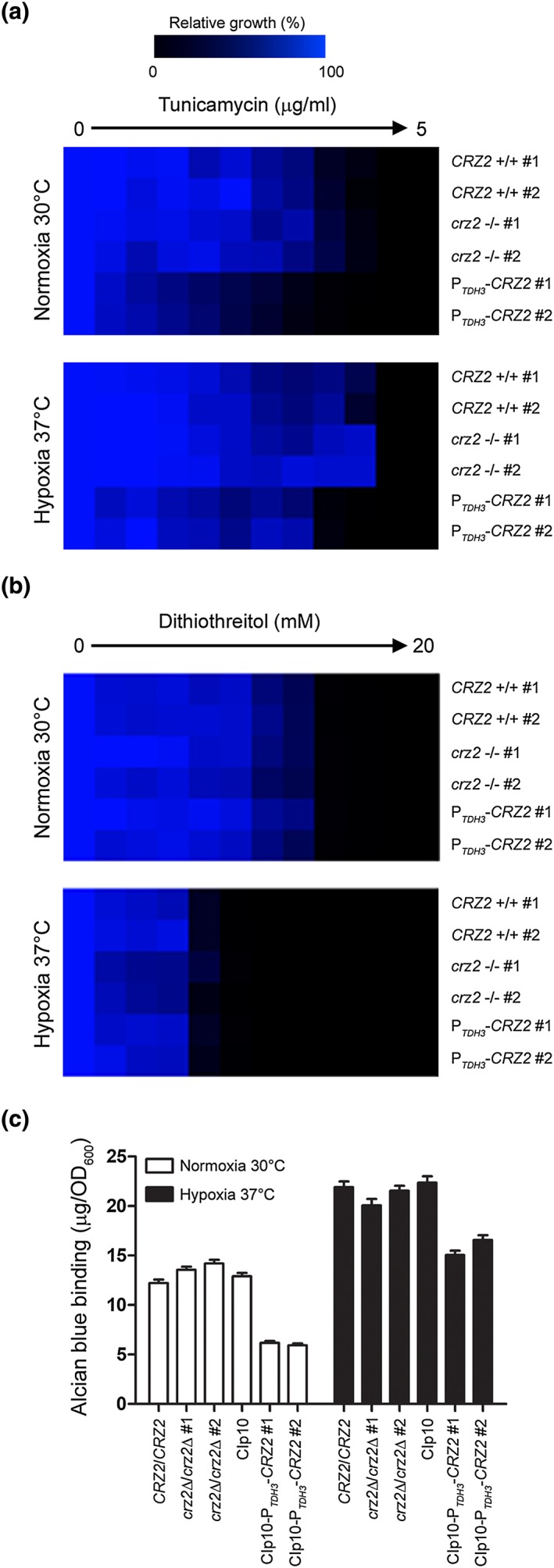
*CRZ2* overexpression interferes with pathways involved in protein glycosylation. (a and b) Tunicamycin (a) and dithiothreitol (b) susceptibilities of two independent parental (*CRZ2* +/+), *CRZ2* deletion (*crz2* −/−), and *CRZ2* overexpression (P_*TDH3*_‐*CRZ2*) strains under normoxia at 30°C and hypoxia at 37°C were determined by microtiter plate assay. The data are presented as the relative growth of the cells in tunicamycin‐ or dithiothreitol‐containing medium (drug concentration range is shown on top of each panel) as compared with growth of the same strain in drug‐free medium. The growth rate values (%) are illustrated using heat maps generated with Genesis version 1.7.6 (Sturn et al., 2002). (c) Alcian Blue binding assay. The reference strain BWP17 (wild‐type control, *CRZ2*/*CRZ2*), two independent *crz2* deletion strains (*crz2*Δ/*crz2*Δ #1 and *crz2*Δ/*crz2*Δ #2) together with strains overexpressing *CRZ2* from the *TDH3* promoter (CIp10‐P_*TDH3*_‐*CRZ2* #1 and CIp10‐P_*TDH3*_‐*CRZ2* #2), and the corresponding parental strain harbouring the empty vector control (CIp10) were grown under normoxia 30°C (open bars) or under hypoxia 37°C (filled bars) and then subjected to Alcian Blue dye binding assay as described in Section 4. Strains are indicated on the *x* axis, and the corresponding Alcian Blue binding values (μg/OD_600_) are indicated on the *y* axis. The assay was performed 3 times independently with averaged values ± standard deviations

Our finding that Crz2p directly regulates the expression of both mannosyltransferase‐ and cell wall‐encoding genes led us to hypothesise that Crz2p could exert its protective role against stresses encountered in the upper GI tract by interfering with pathways affecting protein glycosylation. We tested the susceptibility of the *CRZ2* overexpression strain to the *N*‐glycosylation inhibitor tunicamycin, which blocks *N*‐glycosidic protein‐carbohydrate linkages, using a microtiter plate assay (Figures 10a, see Section 4). Under normoxia 30°C, two independent P_*TDH3*_‐*CRZ2* strains were more susceptible to increasing concentrations of tunicamycin (Figure 10a, upper panel) than was the control or the *crz2*Δ/*crz2*Δ mutant strains. As this phenotype could also be a consequence of tunicamycin‐induced ER stress (e.g., unfolded protein response), we also examined the susceptibility of the P_*TDH3*_‐*CRZ2* strain to another potent inducer of ER stress, dithiothreitol (Zhang, Heitman, & Chen, 2012) and found no difference in growth rate inhibition (Figure 10b, upper panel). Under hypoxia 37°C, overexpression and deletion of *CRZ2* respectively increased and decreased tunicamycin sensitivity (Figure 10a, lower panel). The phenotypes were further validated by spot assay on solid medium containing tunicamycin (Figure S5). No major growth rate difference was observed when strains were grown under the same conditions in the presence of increasing concentrations of the ER stress‐inducing agent dithiothreitol (Figure 10b, lower panel), reinforcing again the specificity of the *CRZ2* phenotype to tunicamycin treatment.

To investigate whether the tunicamycin phenotype correlates with altered glycosylation, we tested the effect of *CRZ2* overexpression on *N*‐glycan outer chain elaboration, by quantifying phosphomannan abundance using cell affinity to Alcian Blue dye (Hobson et al., 2004). We found that *CRZ2* overexpression significantly decreased phosphomannan abundance under both normoxia 30°C and hypoxia 37°C (Figure 10c), unlike the *CRZ2*‐deficient and wild‐type strains. Taken together, our data indicate that *CRZ2* overexpression interferes with pathways involved in protein glycosylation.

## 3 DISCUSSION

Systematic gene overexpression is a powerful approach for linking genotypes to phenotypes and associating genes to biological pathways (Chua et al., 2006; Sopko et al., 2006). Gene overexpression can particularly stimulate a specific activity and mimic gain‐of‐function mutations (Prelich, 2012). Our collection includes 572 strains (near 10% genome‐coverage), each overexpressing one single ORF, using the potent pNIMX tetracycline‐inducible expression system (Chauvel et al., 2012). Our previous study identified only five genes whose overexpression altered C. albicans fitness out of 531 competitively grown signature‐tagged strains, in vitro, using the moderate pNIM1 overexpression system (Cabral et al., 2014). Here, in addition to the previously identified genes (Cabral et al., 2014), we found 21 overexpressers with altered fitness, indicating that increasing overexpression levels correlates with increased sensitivity of our assay. These data also indicate that at least ~10% of the C. albicans genome is still relatively robust to genetic perturbation by gene overexpression, even if we witnessed an increase in the number of hits (5 out of 531 vs. 25 out of 572; Cabral et al., 2014). Consistent with our previous findings, we did not detect genes whose overexpression increased fitness, probably because cells were grown under optimal in vitro growth conditions (YPD, 30°C, normoxia). Our assay also revealed that overexpression of a significant number of genes alters cellular morphology and this appears to translate into a fitness cost (Figure 2a,c). Many of the genes whose overexpression altered cell morphology were also found in our previous screen that analysed both fitness and morphogenesis of individually grown strains, including *RAD53*, *FKH2*, *BEM1*, and *YCK2* (Chauvel et al., 2012). This further validates our competitive assay and our readout approach.

Although 572 overexpression strains were competitively screened in vivo for altered GI tract colonisation, only one hit was found to affect this process: *CRZ2*. This finding was unexpected, knowing that our collection includes a subset of some additional regulators involved in GI tract colonisation, such as *RTG1*, *CPH2*, *LYS144*, and *EFH1* (Table S1). It is possible that the inherent noise generated through animal experimentation prevented the clear detection of these genes in our screen. Alternatively, longer colonisation time (>10 days) might have been required to resolve their overexpression phenotype, and this was shown in a previous loss‐of‐function screen, where the identified hits altered GI colonisation at different time points of stool collection (Perez et al., 2013). For instance, *RTG1* deletion phenotype showed up on Day 9 following strain‐pool inoculation, whereas those of *LYS144* and *EFH1* required extended time (Day 21 and upwards, Perez et al., 2013). As most of the known regulators of GI tract colonisation were identified through loss‐of‐function screens, another explanation is that gene overexpression does not necessarily lead to inversion of the gene‐deletion phenotype (Hoon et al., 2008). We could also have selected for strains with disadvantage in colonisation, including perhaps the fitness defect genes that were already detected in vitro (Figure 2). We believe that, in vitro, cells are under optimal growth in rich medium (with preferential carbon source, complete nutrient broth), which allows the detection of subtle phenotypes. In the gut, however, the different stresses encountered during colonisation and adaptation to those stresses could have masked the effect of the fitness‐defect genes that were seen in vitro.

*CRZ2* encodes a zinc finger TF of the C_2_H_2_ family specifically present among species of the CTG clade that also include commensal species residing in the gut of insects, such as *Pichia stipitis*, *Candida tenuis*, and *Spathaspora passalidarum* (Wapinski, Pfeffer, Friedman, & Regev, 2007). Although Crz2p is homologous to the calcineurin target Crz1p, it is not involved in calcium signalling (Karababa et al., 2006; Kullas et al., 2007), and its function has likely diverged from Crz1p through duplication of a common ancestor (Wapinski et al., 2007). Here, we propose that Crz2p contributes to C. albicans adaptation during early days of GI tract colonisation, because the 1:1 competitive fitness advantage and defect of the *CRZ2* overexpression and deletion strains, respectively, was not maintained during prolonged colonisation (i.e., >4–10 days, Figures 3c,d and S2B,C). A similar observation was reported for Wor1p, the regulator of the GUT phenotype (Pande, Chen, & Noble, 2013), where *WOR1* overexpression increased competitive GI colonisation only within 14–21 days post‐inoculation (Prieto, Roman, Alonso‐Monge, & Pla, 2017). Failure of *WOR1* overexpressers to maintain normal levels of GI colonisation during early days was explained by their increased sensitivity to bile salts (Prieto et al., 2017), suggesting that they are more fit for lower sections of the gut rather than upper sections where bile salts are excreted. Consistent with the hypothesis of preferential colonisation of the upper digestive tract, we show here that *CRZ2* deletion and overexpression respectively increases and decreases C. albicans sensitivity to bile salts and acidic pH (Figure 9), which probably explains the early competitive growth advantage and defect of *CRZ2* overexpression and deletion strains, respectively (Figure 3c,d). This reinforces the notion that different stages of fungal colonisation occur in the mammalian gut environment, as previously described (Prieto & Pla, 2015; Prieto et al., 2017). The complex network of TFs involved in GI tract colonisation that currently includes Wor1p, Efg1p, Efh1p, Cph2p, Lys144p, Tye7p, Rtg1p, Rtg3p, Hms1p, Sfu1p, Zcf8p, Zfu2p, Try4p (Bohm et al., 2017; Chen, Pande, et al., 2011; Perez et al., 2013; Pierce, Dignard, Whiteway, & Kumamoto, 2013; Rosenbach et al., 2010; White et al., 2007), and Crz2p suggests that C. albicans has evolved an extended array of regulators probably acting as relays needed for efficient and sustained growth during early and later processes of C. albicans outgrowth in the GI tract.

We show here that *CRZ2* is transcriptionally induced by hypoxia at 37°C (Figure 8a) and that forced overexpression of *CRZ2* allows to confer resistance to the respiratory chain inhibitor Antimycin A (Figure 7a) in addition to altering reduction of TTC (Figure 7b); reinforcing the notion that the optimal activity of Crz2p occurs under conditions where oxygen is limited. Following a shift to hypoxia at 37°C, the expression of *CRZ2* reached levels (~10‐fold, Figure 8a) similar to those observed upon P_*TET*_‐driven overexpression (approximately eightfold at 4 hr post‐induction, Figure 4a). This indicates that our overexpression system successfully recapitulated the levels attained via physiological activation of Crz2p by hypoxia 37°C, reinforcing the biological relevance of our experimental strategy. How exactly Crz2p adapts to hypoxic conditions awaits further investigations. One of the direct Crz2p targets that may contribute to adaptation to hypoxia and modulation of carbohydrate metabolism is *TYE7* (Figure 6), encoding a basic helix–loop–helix TF involved in transcriptional regulation of glycolytic genes (Askew et al., 2009; Bonhomme et al., 2011). Indeed, Tye7p function was shown to be essential when the respiratory pathway is disrupted, such as in the presence of respiratory chain inhibitors or during growth in a hypoxic environment (Askew et al., 2009). Importantly, Tye7p plays a role in adherence to host cells and favours commensal colonisation (Bohm et al., 2017; Finkel & Mitchell, 2011; Pierce et al., 2013). It is tempting to hypothesise that *TYE7* could be required, at least in part, for Crz2p‐mediated GI tract colonisation.

*CRZ2* is part of a set of adherence regulators required for adhesion of C. albicans to abiotic substrates, some of them modulating the expression of CSTAR (cell‐surface targets of adherence regulators) and HYVIR (hyphal growth or virulence) genes, but *CRZ2* could not be linked to a specific target subgroup (Finkel et al., 2012). One could speculate that *CRZ2* contributes to early gut colonisation by transiently increasing C. albicans adherence to components of the GI environment. Many putative mannoprotein‐encoding genes are direct targets of Crz2p, including *PGA6*, *PGA54*, *PGA38*, *IHD1*, *PHR2*, *PLB1*, *PLB4.5*, *ECM331*, *SAP9*, *UTR2*, *SUN41*, and *INT1* (Figure 6) and carry the *N*‐glycosylation signature Asn‐X‐Ser/Thr in their predicted protein sequence (predicted using NetNGlyc version 1.0). Some of them encode putative adhesins such as *PGA6*, *PGA38*, and *FGR41* or were shown to affect C. albicans adhesion to host cells (*SAP9*, *UTR2*, and *SUN41*; Alberti‐Segui et al., 2004; Albrecht et al., 2006; Hiller, Heine, Brunner, & Rupp, 2007). *INT1* encodes an integrin‐like surface protein required for efficient caecal colonisation in mice (Bendel et al., 2000; Kinneberg et al., 1999); however, the pleiotropic phenotypes of *INT1* make it an unlikely contributor to *CRZ2*‐specific function. At least three Crz2p direct targets (Sun41p, Tos1p, and Scw11p), functioning as secreted β‐1,3‐glucan‐modifying enzymes, were shown to be highly abundant in culture supernatants of C. albicans grown under a variety of growth/stress conditions (Heilmann et al., 2013), suggesting that they rather participate in more general cell‐wall remodelling processes. However, Crz2p direct targets Utr2p, Plb4.5p, and Pir1p become much more abundant upon lowering the environmental pH (Klis & Brul, 2015). Indeed, we show here that *CRZ2* confers survival to highly acidic and oxygen‐scarce environments (Figure 9) similar to those encountered in the stomach. Although the mean intestinal pH in rodents is lower than that in man, the mouse stomach is particularly highly acidic (pH 3 to 4; McConnell, Basit, & Murdan, 2008). Crz2p directly controls the expression of *PHR2* (Figure 6), encoding a cell‐wall β‐glycosidase that is expressed at acidic pH (Muhlschlegel & Fonzi, 1997) and was shown to be required for virulence in a rat model of vaginal infection (De Bernardis, Muhlschlegel, Cassone, & Fonzi, 1998). Many Crz2p direct targets were also expressed at the C. albicans cell wall under hypoxia at 37°C in a vagina‐simulative medium (Sosinska et al., 2008) as well as in an in vitro system that mimics colonisation of mucosal surfaces at acidic pH, including *PHR2*, *ECM331*, *PIR1*, *UTR2*, and *TOS1* (Sosinska et al., 2011), further reinforcing the notion that Crz2p is an important contributor to C. albicans ability to grow in low‐pH environments.

Candida albicans adaptation to the mammalian gut is tightly linked to nutritional requirements, such as the need for preventing iron toxicity (Chen, Blyth, Sorrell, & Slavin, 2011), the use of amino acids/allantoate and carbohydrates as a source of energy (Perez et al., 2013), and the catabolism of fatty acids/*N*‐acetylglucosamine or phosphate uptake (Pande et al., 2013; Pierce et al., 2013). It is also linked to the ability of preventing bile‐salt toxicity, particularly in the upper section of the gut. *CRZ2* confers increased resistance to bile salts (Figure 9), which is considered a major selective pressure that shapes the structure of the microbial communities residing in the gut (Ridlon, Harris, Bhowmik, Kang, & Hylemon, 2016). Many bacterial species that colonise the GI tract play a role in bile salt metabolism and are able to make bile salts available as substrates for further modifications by the intestinal microbiota, mainly through the activity of bile salt hydrolases (Urdaneta & Casadesus, 2017). Our inspection of the fungal genomes suggests that the genome of only one *Candida* species, *Candida kefyr*, encodes a putative bile salt hydrolase (GenBank accession # AFC60678.1), whose BLAST analysis against the C. albicans proteome did not return any significant hit. Bile‐salt resistance is also conferred by the expression of multidrug efflux pumps and alteration of membrane lipid/protein composition (Ridlon et al., 2016). At least 15 Crz2p direct targets encode membrane transporters (Figure 6), two of which are members of the major facilitator superfamily: *QDR3* and orf19.7554. Qdr3p is similar to Escherichia coli MdtM, a major facilitator superfamily drug/H^+^ antiporter of the DHA1 subfamily that confers bile‐salt resistance through catalysis of electrogenic bile salt/H^+^ antiport (Paul et al., 2014). Whether *QDR3* confers Crz2p‐mediated resistance to bile salts awaits further investigation. As *CRZ2* genetic perturbation could affect cell‐wall integrity, one could speculate that it may also alter the composition of membrane lipids and proteins with the consequence of decreasing C. albicans susceptibility to bile salts. We propose that the combined action of the hypoxic environment within the GI tract and the required adaptation to low pH and bile‐salt toxicity in the upper digestive tract are crucial for *CRZ2*'s role in C. albicans ability to thrive within the gut. Our findings and those already reported by others suggest that C. albicans adaptation to the gut is a complex process requiring tight and combined control of sensing abilities, extended morphological and metabolic activities, and specific adaptive responses.

## 4 EXPERIMENTAL PROCEDURES

### 4.1 Candida albicans strains

The C. albicans signature‐tagged overexpression collection was constructed as described previously (Cabral et al., 2014; Chauvel et al., 2012; see Table S1 for a list of all ORFs included and corresponding barcode sequences and Table S11 for additional strains used in this study). Briefly, the respective ORFs were PCR amplified using previously designed chimeric primers (Cabral et al., 2014) followed by recombination‐mediated transfer into the Gateway donor vector pDONR207 (Invitrogen). The set of pDONR207 derivatives was fully sequenced to ascertain that no unintended mutations were introduced during PCR amplification. The pDONR207‐ORF plasmids were then used in a Gateway LR reaction together with barcoded derivatives of the CIp10‐P_*TET*_‐GTW vector (Cabral et al., 2014; Chauvel et al., 2012). All barcoded overexpression vectors were linearised with *Stu*I and used to transform strain CEC2908, a derivative of the BWP17AH strain that carries the pNIMX plasmid (isogenic to CEC2907 described in Chauvel et al., 2012, Table S1). Transformants were selected and checked by PCR for correct integration yielding 572 overexpression strains (Table S1). Seven strains carried altered barcode sequences and were therefore omitted from the microarray analyses (Table S1, strains marked in red). The barcoded CIp10‐P_*TET*_‐*CRZ2* or CIp10‐P_*TET*_‐GTW control plasmids were used to transform strains CEC3783 or CEC3781 (Cabral et al., 2014) carrying the pNIMX plasmid for doxycycline‐regulated expression from the P_*TET*_ promoter and either a P_*TDH3*_ *‐*BFP or P_*TDH3*_ *‐*GFP gene fusion for constitutive expression of BFP or GFP, respectively, generating strains CEC4442 or CEC4439, respectively (Table S11).

The C. albicans *crz2*Δ/*crz2*Δ mutants were generated in the BWP17 (*ura3*, *arg4*, and *his1* auxotroph) background by successive replacement of the complete ORF from both alleles using PCR‐generated disruption cassettes flanked by 100 bp of target homology region as previously described (Gola, Martin, Walther, Dunkler, & Wendland, 2003). The disruption cassettes were amplified using oligonucleotides CRZ2_F_KO and CRZ2_R_KO (see Table S12 for primers used in this study) and *ARG4‐* or *HIS1‐*bearing plasmids. The resulting transformants were verified by PCR, and two independent clones were selected for subsequent transformation with plasmid CIp10 or plasmid CIp10‐P_*TDH3*_‐GFP yielding the prototrophic *crz2*Δ/*crz2*Δ mutants C89 and C90 (two independent CIp10 plasmid integrants) or CEC4263 and CEC4265 (CIp10‐P_*TDH3*_‐GFP integrants that constitutively express GFP), respectively (Table S11). Strain CEC155 (Table S11; Firon et al., 2007) was first transformed with a PCR‐amplified DNA fragment carrying both P_*TDH3*_‐BFP fusion and *HIS1* marker such that the whole cassette integrates between the *PGA62* and *PGA59* loci, as previously described (Cabral et al., 2014). The resulting strain was transformed with plasmid CIp10 to generate the prototrophic strain CEC4425 (Table S11) that we used as a BFP‐labelled control for our 1:1 competitive assays (Figure 3c,d). Strains overexpressing *CRZ2* from the constitutive *TDH3* promoter were generated by transferring the *CRZ2* ORF from the respective pDONR207 derivative to the Gateway‐compatible CIp10‐P_*TDH3*_‐GTW plasmid (Legrand et al. 2018) followed by transformation of strain BWP17AH (CEC161; Chauvel et al., 2012) to generate three independent P_*TDH3*_‐driven *CRZ2*‐overexpression clones (C26, C27, and C28; Table S11). The CEC161 strain was also transformed with CIp10‐P_*TDH3*_‐GFP, generating strain CEC4267 (Table S11), to serve as a control for phenotypic assays.

To create a TAP epitope‐tagged version of *CRZ2*, the *CRZ2* ORF was transferred from the corresponding pDONR207 to the Gateway‐compatible CIp10‐P_*TET*_‐TAP‐GTW that allows P_*TET*_‐driven expression of N‐terminally‐tagged ORFs (Legrand et al., 2018, in press). The *ura3* auxotrophic derivative of the *crz2*Δ/*crz2*Δ mutants was first transformed with plasmid pNIMX then with CIp10‐P_*TET*_‐TAP‐*CRZ2* or CIp10‐P_*TET*_‐*CRZ2* (untagged control) to generate strains C251, C252 (two independent TAP‐tagged clones) and C255, C257, C258 (three independent untagged control clones), respectively (Table S11). Expression of the TAP‐Crz2p fusion was confirmed by Western blotting as described in Znaidi et al. (2013).

### 4.2 Preparation of strain pools

The 572 signature‐tagged overexpression strains were thawed on Nunc omnitray plates (Thermo Scientific) containing YPD (1% Yeast Extract, 2% Bacto‐Peptone, 2% D‐glucose)‐agar supplemented with 50 μg ml^−1^ gentamycin using a 96 pin replicator and allowed to grow for 4 days at 30°C. No major colony size alterations were noticed. Five millilitres of YPD were added to each plate, and colonies were scraped off using a cell spreader. Strains were pooled in ~100 ml YPD/15% glycerol at a concentration of ~50 or ~132 OD_600_ (optical density at 600 nm) units/ml, aliquoted in 1.5‐ml tubes and frozen at −80°C.

### 4.3 In vitro competitive fitness assay

An aliquot (1.6 μl) from the frozen 50 OD_600_‐unit strain‐pool was used to inoculate 2 ml of YPD medium (starting OD_600_ = 0.0625) and grown at 30°C with agitation (200 rpm) for 20 generations, in the absence or presence of 40 μg ml^−1^ doxycycline. Genomic DNA was extracted using the MasterPure Yeast DNA Purification Kit (Epicentre) and quantified using a NanoVue Plus device (GE Healthcare Life Sciences). Barcodes were PCR‐amplified using primers CipSAC2‐UP‐2 and CipSAC2‐DWN‐2 (3 min at 94°C; followed by 35 cycles of 30 s at 94°C, 30 s at 50°C, and 30 s at 72°C; and a final step of 7 min at 72°C; Table S12). The PCR products were then purified and subjected to indirect differential fluorescent dye labelling (Cy5 for Dox‐treated, Cy3 for untreated pools). Labelled DNA was resuspended in 50 μl DigEasy Hyb solution (Roche), incubated at 95°C for 5 min, snap‐cooled on ice, and directly deposited on a barcode microarray that we previously described (Agilent Technologies, GEO platform # GPL17420; Cabral et al., 2014) containing (a) ~12 on‐chip replicates of both sense and antisense DNA sequences complementary to 657 tags (representing 572 strain tags +78 unused tags) and (b) different negative control spots (Agilent reference). Hybridization was performed overnight at 25°C, followed by washing and scanning of the arrays using GenePix 4200 AL scanner (Molecular Devices). This experiment was performed 3 times independently. Microarray data were analysed using ArrayPipe v2.0 (Hokamp et al., 2004). *Z*‐score (i.e., number of standard deviations from the population mean) calculations were performed using ArrayPipe v2.0, and thresholds for considering significant deviation from the population were set at absolute *Z*‐score values ≥2 and *P* values <0.05. Only strains with tags that met thresholds for both sense‐ and antisense‐barcode fluorescence signals were considered as altered in cell fitness. Microarray data have been deposited at GEO under accession number GSE67215, and *Z*‐scores and fold‐change data are available in Table S2.

### 4.4 Confirmation of the in vitro fitness‐profiling data by liquid growth assay or microscopic examination

Strains were individually grown 3 times independently in 96‐well plates at a starting optical density (OD_600_) of 0.1 in 100 μl of YPD supplemented or not with 40 μg ml^−1^ doxycycline. The OD_600_ was measured every 5 min using a TECAN Infinite M200 reader. The temperature was set at 30°C. TECAN OD_600_ readings were converted into “flask OD_600_” reading using the following formula: OD_Flask_ = OD_Tecan_ × 12.2716 – 1.0543 (Ericson, Hoon, St Onge, Giaever, & Nislow, 2010), and doubling times were calculated within the exponential growth interval as previously described (St Onge et al., 2007). Strains displaying altered morphology or cell–cell aggregation phenotypes were microscopically examined with a Leica DM RXA microscope (Leica Microsystems) at a 40× magnification. Images were captured with a Hamamatsu ORCA II‐ER cooled CCD camera, using the Openlab software version 3.5.1 (Improvision Inc.).

### 4.5 In vivo competitive GI tract colonisation screen

All animal experiments adhered to the EU Directive 86/609 on the approximation of laws, regulations, and administrative provisions of Member States regarding the protection of animals used for experimental and other scientific purposes and to related national regulations. All experiments were performed according to the guidelines of the European Convention for the Protection of Vertebrate Animals used for Experimental and Other Scientific Purposes (ETS # 123). The protocol was approved by Institut Pasteur Health Center Animal Care Committee (protocol number 10.455). Nine‐ to 12‐weeks‐old female BALB/c (Charles River, France) were given drinking water containing 5% sucrose, 0.1 mg ml^−1^ gentamycin, and 2 mg ml^−1^ streptomycin and was supplemented or not with 2 mg ml^−1^ doxycycline, during the course of the whole experiment (14 days). Mice were housed by groups of three to five individuals per cage and were inoculated by gavage with 5 × 10^7^ C. albicans cells (in 200 μl 1X phosphate‐buffered saline [PBS] buffer) from 572‐strain pools at Day 4 post‐antibiotic treatment. The remaining inoculum was directly used for genomic DNA extraction using the MasterPure Yeast DNA Purification Kit (Epicentre). Stool samples were collected at Day 10 post‐gavage, weighed, and either (a) homogenised then serially diluted in 1 ml 1X PBS (2 stool pellets) for CFU counting on YPD plates supplemented with 1 g L^−1^ chloramphenicol and 50 mg L^−1^ gentamycin (YPDCG) or (b) directly processed (up to 500 mg of stool pellets) for genomic DNA extraction using FastDNA SPIN Kit for Faeces according the manufacturer's instructions (MP Biomedicals, cat. # 6570). Colonisation efficiency was within the range of ~10^6^ to ~10^8^ CFUs per 1 g of stool on Day 10. Barcodes were directly amplified from dox‐treated (Cy5‐labelled), dox‐untreated (Cy5‐labelled) mouse stool‐derived, and inoculum‐derived (Cy3‐labelled) genomic DNA preps using primers CipSAC2‐UP‐2 and CipSAC2‐DWN‐2 and processed for hybridization to barcode microarrays as described above (Section 4.3). Data were analysed as described above (Section 4.3). Microarray data have been deposited at GEO under accession number GSE67215, and *Z*‐scores and fold‐change data are available in Tables S3 and S4. An overview of the design of the competitive fitness profiling experiment in mice is shown in Figure S1.

### 4.6 Validation of the GI tract colonisation screen

Q‐PCR assays were performed to quantify the relative abundance of strains carrying P_*TET*_‐*CRZ2* (test), P_*TET*_‐*PGA37*, P_*TET*_‐orf19.3088, P_*TET*_‐*CNB1*, and P_*TET*_‐*IHD1* (controls) using genomic DNA extracted from pooled stools at Day 10 post‐gavage from two independent dox‐treated and dox‐untreated cages (each housing three mice) relative to the inoculum. Forward primers CRZ2‐BC, PGA37s‐BC, orf19.3088‐BC, CNB1‐BC, and IHD1‐BC (Table S12) complementary to the 20‐bp barcode sequence of the corresponding strains were used in combination with the reverse primer CIPSAC2‐DWN3 (Table S12), which hybridizes 66 bp downstream of each barcode sequence, to amplify a 106‐bp amplicon in up to 7 independent 20 μl‐Q‐PCR reactions containing 1 μl genomic DNA (initially, different dilutions were tested for efficient amplification), 4 μl of primer mix at 10 pmol μl^−1^ each, 10 μl of 2X Takyon Rox SYBR MasterMix dTTP Blue (Eurogentec), and 5 μl of H_2_O. Reactions were processed in a MicroAmp Optical 96‐Well Reaction Plate (Applied Biosystems) using an Eppendorf *realplex* ^*4*^ Mastercycler real‐time PCR instrument (Eppendorf) with 1 cycle at 50°C for 2 min, 1 cycle at 95°C for 10 min, and 50 cycles at 95°C for 15 s and 58°C for 1 min, followed by melting‐curve generation to rule out amplification of unspecific products. Data were analysed using the *realplex* software version 2.2 (Eppendorf). For each experiment, threshold cycle (C_T_) values were determined using the *realplex* software. The relative abundance (n‐fold) of each strain from dox‐supplemented or dox‐free cages as compared with the inoculum was calculated using the 2^−ΔΔCT^ method, as follows: ΔC_T_ = C_T_ (selected strain) − C_T_ (*TEF3* reference for calibration) in each condition and ΔΔC_T_ = ΔC_T_ (dox − treated or dox − untreated sample) − ΔC_T_ (inoculum). The *TEF3* gene was used for calibration, and the *ACT1* gene was used as a control for normalisation of C. albicans DNA abundance (primers TEF3‐F/R and ACT1‐F/R, Table S12). Up to seven independent qPCR experiments were performed on different days using two technical replicates each time. A two‐tailed Student's *t* test was applied by comparing the relative abundance of each strain from doxycycline‐treated cages to the untreated controls. Statistical significance was set at *P* < 0.05. Data from only one cage are shown in Figure 3b and are representative of those from the second cage.

To independently validate the *CRZ2* overexpression phenotype and test the effect of *CRZ2* deletion on GI colonisation, 5 × 10^7^ cells from a 1:1 mixture of CEC4439 (P_*TET*_‐*CRZ2*, GFP):CEC4442 (P_*TET*_‐empty vector, BFP), CEC4267 (wild‐type, GFP):CEC4425 (wild‐type control, BFP), CEC4263 (*crz2*Δ/*crz2*Δ mutant #1, GFP):CEC4425 (wild‐type control, BFP), or CEC4265 (*crz2*Δ/*crz2*Δ mutant #2, GFP):CEC4425 (wild‐type control, BFP) in 200 μl 1X PBS were used to inoculate BALB/c or BALB/cByJ mice as described above. The remaining inoculums were analysed by flow cytometry with a MACSQuant (Miltenyi Biotec) flow cytometer to quantify the relative abundance of Gfp^+^ and Bfp^+^ cells and confirm the near 1:1 distribution of the signals (see below). For the 1:1 competition using the P_*TET*_ promoter, doxycycline at a final concentration of 2 mg ml^−1^ was added (test) or not (control) to the drinking water of mice as described above. Stools were collected at Days 4, 10, and 14 post‐gavage, homogenised and then serially diluted in 1 ml of 1X PBS (2 stool pellets), and plated on YPDCG during 2 days at 30°C. Selected dilutions from the resulting CFUs (typically 400 to 4,000 CFUs) were scraped off using a cell spreader after addition of 5 ml 1X PBS buffer. The resulting pooled cells were thoroughly vortexed and diluted 1:1,000 in 1X PBS and then analysed with the MACSQuant flow cytometer. We used the 405‐ and 488‐nm lasers to excite the BFP and GFP proteins (whose expression was driven by the *TDH3* promoter) and the 425/475 and 500/550 filters to detect the BFP and GFP emission signals. Flow cytometry outputs were analysed with FlowJo 7.6. Gates that define the Bfp^+^ and Gfp^+^ populations were created with one of the control samples (dox‐untreated CEC4439:CEC4442 or CEC4267:CEC4425) and applied to the remaining samples. The percentage values of Bfp + (%BFP) Gfp + (%GFP) cells were exported to Excel spreadsheets and further processed. CIs were determined by dividing the ratio of %GFP (P_*TET*_‐*CRZ2*, wild‐type or *crz2*Δ/*crz2*Δ mutants) and %BFP (P_*TET*_‐empty vector, wild‐type control) strains on Days 4, 10, or 14 by the ratio in the inoculum, that is, [(%GFP/ % BFP) at Days 4, 10, or 14]/[(%GFP/ % BFP) in the inoculum] as previously described (White et al., 2007). Competitive assays using P_*TET*_ were performed up to 4 times independently totalling *n* = 5 to 12 dox‐untreated mice and *n* = 5 to six dox‐treated animals (Figures 3c and S2B, six dox‐treated mice showed signs of illness and were removed from the analysis). Two reproducible competition assay experiments were performed for the *crz2*Δ/*crz2*Δ mutants #1 and #2 versus wild‐type strain (*n* = 5 mice in each condition, one representative experiment is shown in Figures 3d and S2C). A two‐tailed non‐parametric Mann–Whitney test was used for assigning statistical significance (*P* < 0.05).

### 4.7 Whole‐genome transcript profiling experiments

For the P_*TET*_‐*CRZ2* microarray experiment (Figure 4), total RNA was extracted from three independently generated *crz2*Δ/*crz2*Δ strains carrying the P_*TET*_‐*CRZ2* fusion (C255, C257, and C258; Table S11), pre‐grown overnight in 10 ml YPD at 30°C and then diluted in fresh YPD medium supplemented or not with 40 μg ml^−1^ doxycycline to an OD_600_ of 0.3 and regrown for 2 and 4 hr. For the normoxia 30°C‐to‐hypoxia 37°C shift microarray experiments (Figure 8), strains CEC369 (WT) and C90 (*crz2*Δ/Δ; Table S11) were grown overnight in 10 ml YPD at 30°C and then diluted to an OD_600_ of 0.16 in 250‐ml flasks containing 50 ml of YPD medium. The flasks were incubated in a BBL GasPak anaerobic jar at 37°C for 24 hr without shaking (hypoxia 37°C) or in a 30°C incubator for 24 hr under vigorous shaking (normoxia 30°C). Total RNA was extracted from 50 OD units using the hot phenol method as described previously (Znaidi et al., 2013), followed by first‐strand cDNA synthesis and Cy5 (dox‐treated cDNA samples for the P_*TET*_‐*CRZ2* experiment and hypoxia 37°C‐treated cDNA samples for the normoxia 30°C‐to‐hypoxia 37°C shift experiment)/Cy3 (untreated cDNA samples for the P_*TET*_‐*CRZ2* experiment and normoxia 30°C‐treated cDNA samples for the normoxia 30°C‐to‐hypoxia 37°C shift experiment) labelling from 20 μg total RNA, using the SuperScript III indirect cDNA labelling system (Invitrogen). Purified labelled samples were mixed and hybridized to a C. albicans expression array (Agilent Technologies) designed such that two nonoverlapping probe sets target each of 6,105 C. albicans ORFs from Assembly 19 for a total of 15,744 probes, thereby allowing two independent measurements of the mRNA level for a given gene (Znaidi et al., 2013). As an additional control experiment, we also compared the transcriptome of strain C90 (Cy5‐labelled) to that in *crz2*Δ/Δ (CEC369, Cy3‐labelled) under normoxia 30°C and under hypoxia 37°C (Table S6). Hybridization was performed as described elsewhere (Znaidi et al., 2013). Images of the Cy5 and Cy3 fluorescence were generated by scanning the expression arrays using an Axon Autoloader 4200AL scanner (Molecular Devices, Downingtown, PA, USA). Images were analysed with the GenePix Pro 6.1.0.2 software (Molecular Devices). GenePix Results files were imported into the ArrayPipe 2.0 software for spot filtering, background subtraction (limma normexp BG correction), and Lowess global normalisation of signal intensities (Hokamp et al., 2004). Replicate arrays (*n* = 3) were combined, and fold‐change and *P* values (standard Student's *t* test within group) were calculated. The complete expression profiling datasets are available in Table S6. Expression microarray data have been deposited at GEO under accession number GSE67226.

### 4.8 Chromatin immunoprecipitation and ChIP‐chip

Strains C251, C252 (TAP epitope‐tagged), and C255 and C257 (untagged control; Table S11) were grown overnight in 2 ml YPD at 30°C, diluted to an OD_600_ of 0.3 in 50 ml YPD medium supplemented with 40 μg ml^−1^ doxycycline and grown during 4 hr at 30°C. The subsequent steps of DNA cross‐linking, DNA shearing, and chromatin immunoprecipitation (ChIP) were conducted as described in Liu et al. (2007), with some modifications. Briefly, cultures were treated with 1% formaldehyde (cross‐linking) and snap‐frozen in liquid nitrogen. Total cell extracts were prepared by bead beating using a FastPrep‐24 instrument (MP Biomedicals) with 10 runs during 40 s each at 5.5 m s^−1^ and 1 min on ice in between. Soluble chromatin fragments were prepared by sonicating the extracts 6 times during 20 s at power 8 (knob position) for an output signal amplitude of 15 (Microns, Peak to Peak) using a probe sonicator (MSE), yielding a majority of ~200–500 bp DNA fragments. Immunoprecipitation was conducted overnight at 4°C with 500 μl of clarified sonicated extracts and 40 μl of IgG‐coated magnetic beads (Dynabeads Pan mouse IgG, Invitrogen), previously pre‐hybridized overnight with PBS‐0.1% BSA at 4°C. The concentration of the purified immunoprecipitated (IP) DNA ranged between 31 and 147 pg μl^−1^ in 50 μl TE (10 mM Tris [pH 8.0], 1 mM EDTA). DNA was labelled as described by Drouin and Robert (http://www.ircm.qc.ca/LARECHERCHE/axes/Biologie/Chromatine/Documents/ProtocoleIRCM_LevureYeast1.pdf). Briefly, the IP DNA (40 μl out of 50 μl) fragments were blunted with T4 DNA polymerase and ligated to unidirectional linkers. The DNA was amplified by ligation‐mediated PCR in the presence of aminoallyl‐modified dUTP. The labelling was carried out post‐PCR using monoreactive Cy dye *N*‐hydroxysuccinimide esters (Cy5/Cy3 monoreactive dye packs; Amersham Biosciences) that react specifically with the aminoallyl‐modified dUTP [5‐(3‐aminoallyl)‐2′ deoxyuridine‐5′‐triphosphate; Sigma‐Aldrich]. Labelled IP DNA from the tagged C251 and C252 strains (Cy5) and the untagged control strains (C255 and C257, Cy3) were mixed and hybridized to previously described C. albicans whole‐genome tiled DNA microarrays from Assembly 20 (Tuch, Galgoczy, Hernday, Li, & Johnson, 2008; GEO platform accession # GPL13696). Images of Cy5 and Cy3 fluorescence intensities were generated by scanning arrays using an Axon Autoloader 4200AL scanner and analysed with the GenePix software version 7.0 (Molecular Devices). Data normalisation (Quantile) and peak finding were conducted using CisGenome (Ji et al., 2008). The Integrated Genomics Viewer software was used for visualisation of the ChIP‐chip results (Thorvaldsdottir, Robinson, & Mesirov, 2012; Figure 5a). The complete Crz2p binding datasets are available in Tables S8–S10. ChIP‐chip raw data were deposited at GEO under accession number GSE67233.

### 4.9 Validation of transcriptomics and ChIP‐chip experiments

Total RNA from strains C255, C257, and C258 (P_*TET*_‐*CRZ2* microarray experiment) or strains CEC369 and C90 (normoxia 30°C‐to‐hypoxia 37°C microarray experiment) was extracted using the hot phenol method and reverse‐transcribed (5 μg of total RNA) using the SuperScript III first‐strand synthesis system (Invitrogen, catalogue # 18080‐051) in a total reaction volume of 20 μl. The qPCR reactions were made of 1 μl from the RT reaction mixture (or diluted RT reaction, when required, to optimise amplification efficiency) combined with 4 μl of primer mix at 10 pmol μl^−1^ each (forward and reverse primers of the selected genes, Table S12), 10 μl of 2X Takyon Rox SYBR MasterMix dTTP Blue (Eurogentec), and 5 μl of H_2_O. qPCR reaction conditions are described above (Section 4.6). For the P_*TET*_‐*CRZ2* experiment (at 2 and 4 hr time points), levels of relative gene expression (n‐fold, for the doxycycline‐treated samples as compared with the untreated controls) of *CRZ2* (primers Crz2_F_qPCR_2 and Crz2_R_qPCR_2), *MNN1* (MNN1‐F/R), *PGA6* (PGA6‐F/R), *ECM331* (ECM331‐F/R), *PLB1* (PLB1‐F/R), *RHD3* (RHD3‐F/R), *PHO84* (PHO84‐F/R), *RHD1* (RHD1‐F/R), and the *ACT1* (ACT1‐F/R) negative control gene (Figure S4) were calculated using the 2^−ΔΔCT^ method, as follows: ΔC_T_ = C_T_ (selected gene) − C_T_ (*TEF3* reference gene), calculated for both dox‐treated and untreated samples, and ΔΔC_T_ = ΔC_T_ (doxycycline − treated sample) − ΔC_T_ (untreated sample). For the normoxia 30°C‐to‐hypoxia 37°C experiment, levels of relative gene expression (n‐fold, for the hypoxia 37°C samples as compared with the normoxia 30°C samples in strains CEC369 or C90) of *MNN1* (MNN1‐F/R), *UTR2* (Utr2‐RT‐f and Utr2‐RT‐r), *PHO84* (PHO84‐F/R), *RHD1* (RHD1‐F/R), and the *ACT1* (ACT1‐F/R) negative control gene (Figure 8b) were calculated using the 2^−ΔΔCT^ method, as follows: ΔC_T_ = C_T_ (selected gene) − C_T_ (*TEF3* reference gene), calculated for both hypoxia 37°C and normoxia 30°C samples, and ΔΔC_T_ = ΔC_T_ (hypoxia 37^°^C sample) − ΔC_T_ (normoxia 30^°^C sample). For the ChIP‐qPCR experiment, the levels of target‐DNA enrichment (n‐fold, Table S12 lists primer sequences) for *CRZ2* (primers CRZ2prom‐F/R), *MNN1* (MNN1prom‐F/R), *ECM331* (ECM331prom‐F/R), *MNN22* (MNN22prom‐F/R), *RHD1* (RHD1prom‐F/R), *RHD3* (RHD3prom‐F/R), and *ACT1* (ACT1‐F/R, used as a negative control) were calculated using relative quantification according to the 2^−ΔΔCT^ method, as follows: ΔC_T_ = C_T_ (target) − C_T_ (*TEF3* reference), calculated in both tagged (TAP‐Crz2p, strains C251 and C252) and untagged (untagged Crz2p, strains C255 and C257) samples, and ΔΔC_T_ = ΔC_T_ (tagged) − ΔC_T_ (untagged), where C_T_ (*TEF3* reference) is the C_T_ for the *TEF3* amplicon (primers TEF3‐F/R, Table S12). Assays were performed (a) at least 2 times using three biological replicates for the qRT‐PCR experiments or (b) 3 times independently with two biological replicates each time. A two‐tailed Student's *t* test was applied by comparing, for a given gene/ChIP target, the n‐fold relative gene‐expression values to the corresponding n‐fold values of the *ACT1* control (Figures S4 and 8b) or, for ChIP‐qPCR, the n‐fold enrichment values of the selected target gene (*CRZ2*, *MNN1*, *ECM331*, *MNN22*, *RHD1*, or *RHD3*) to those of the corresponding *ACT1* control (Figure 5b). Statistical significance threshold was *P* < 0.05.

### 4.10 Alcian Blue binding of cell wall mannans

Alcian Blue binding assay was performed as described in Odani, Shimma, Tanaka, and Jigami (1996). Briefly, cells were grown in YPD medium at 30°C for 48 hr or at 37°C for 48 hr under hypoxic conditions and then harvested by centrifugation, washed with 0.02 M HCl, and resuspended in 1 ml of 100 μg ml^−1^ Alcian Blue HCl solution (Merck Millipore, Germany). After incubation at room temperature for 10 min, cells were pelleted, and absorbance (OD_600_) of the supernatant was determined in a microplate reader. Alcian Blue binding was calculated following the formula: (μg/OD600) = 61.3 × (OD_600_ original solution − OD_600_ supernatant)/OD_600_ cell density.

### 4.11 Spot and microtiter plate assays

For spot assays, cell patches from strains CEC369 (*CRZ2*/*CRZ2*, control), C89‐C90 (*crz2*Δ/*crz2*Δ), C74 (CIp10, control), C26‐C27 (two independently generated CIp10‐P_*TDH3*_‐*CRZ2* strains), CEC4442 (CIp10‐P_*TET*_‐GTW, control), and CEC4439 (CIp10‐P_*TET*_‐*CRZ2*; Table S11) were grown overnight in YPD medium and resuspended in water to an optical density at 600 nm of 0.1. Tenfold serial dilutions of each strain were spotted onto (a) SD or YPD plates supplemented with 20 μg ml^−1^ Antimycin A (from *Streptomyces* sp., A8674‐50MG, Sigma Aldrich, +/− 3 μg ml^−1^ aTc) or 2 μg ml^−1^ of tunicamycin (from *Streptomyces* sp., T7765‐5MG, Sigma Aldrich) or (b) SD plates supplemented with 150 mM HEPES (Gibco, Invitrogen) buffered at pH 3 or supplemented with 0.1% bile salts (48305‐50G‐F, Sigma Aldrich). The plates were incubated for 1–3 days at 30°C or 37°C under normoxic conditions or for 3–7 days at 30°C or 37°C in a BBL GasPak anaerobic jar. For the microtiter plate assays, cells were grown overnight in YPD medium and then diluted in YPD to an OD_600_ of 0.1, followed by a second 1:100 dilution in the same medium. Fifty microlitres of this dilution was added to 50 μl of a tunicamycin or dithiothreitol solution (in YPD) concentrated 2 times or drug‐free medium in 96‐well microtiter plates. The range of drug concentrations tested was 0.15, 0.25, 0.31, 0.50, 0.62, 1.00, 1.25, 2.00, 2.50, 4.00, and 5.00 μg ml^−1^ of tunicamycin and 0.62, 1.09, 1.25, 2.19, 2.50, 4.37, 5.00, 8.75, 10.00, 17.50, and 20.00 mM of dithiothreitol. Growth was measured spectrophotometrically at OD_600_ after 24 hr of incubation at 30°C in a humid chamber or after 4 days under hypoxia at 37°C.

### 4.12 Bioinformatic analyses

GSEA were performed as described previously (Sellam et al., 2014). Briefly, the GSEA PreRanked tool (http://www.broadinstitute.org/gsea) was used (default parameters) to determine if our lists of ranked genes (P_*TET*_‐*CRZ2* data at 2 and 4 hr sorted from the highest upregulated genes to the highest downregulated ones) display a significant bias with any gene sets from a compendium of published microarray data, GO term categories/pathways, and data derived from S. cerevisiae resources (Sellam et al., 2014). The Cytoscape EnrichmentMap plugin was used to further visualise the GSEA network using the default parameters (Figure 4b,c). For motif discovery analyses, peak summit location files generated by CisGenome (Tables S8 and S9; Ji et al., 2008) were imported into the Galaxy NGS analysis pipeline (https://main.g2.bx.psu.edu/), and DNA sequences encompassing ±250 bp around peak summits in Crz2p datasets were extracted using the Extract Genomic DNA tool version 2.2.2. The resulting sequences together with an equivalent set of randomly chosen sequences from the C. albicans genome (used as a negative control) were used as input for motif discovery with the Regulatory Sequence Analysis Tools (http://rsat.ulb.ac.be/rsat) peak‐motifs algorithm (Thomas‐Chollier et al., 2012). Significance coefficients of overrepresented motifs (*sig* scores =  − log_10_ [E − value]) are calculated using a binomial test.

## Supporting information

Supporting info item

Supporting info item

Supporting info item

Supporting info item

Supporting info item

Supporting info item
