# Supplementary figures and images for: Systematic gene overexpression in Candida albicans identifies a regulator of early adaptation to the mammalian gut
Source: Cell Microbiol. 2018 Aug 7;20(11):e12890. doi: 10.1111/cmi.12890 (PMC6220992; doi:10.1111/cmi.12890)

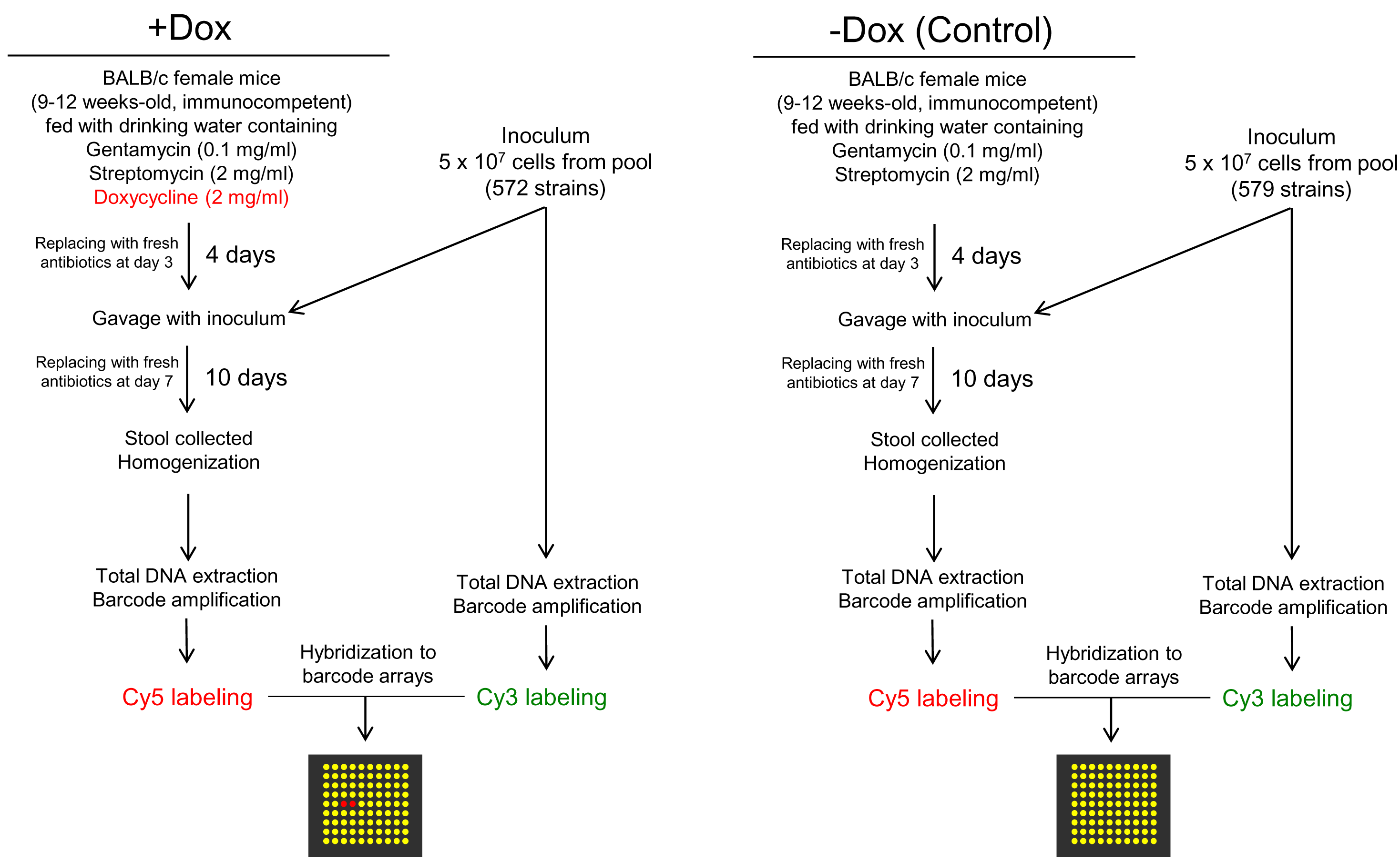

Supplement: Supplementary file 1 — Supporting info item [file CMI-20-na-s001.tif]

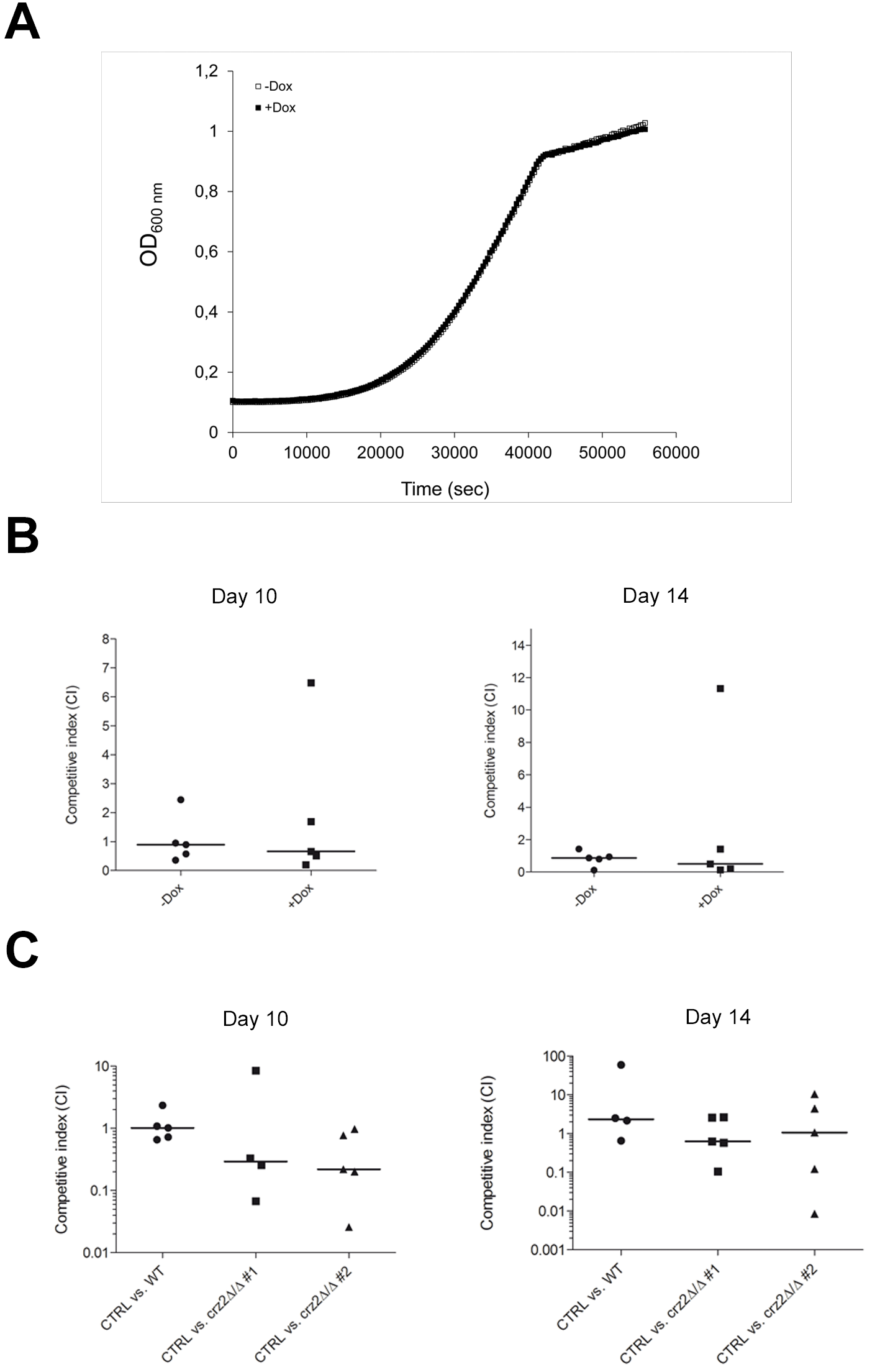

Supplement: Supplementary file 2 — Supporting info item [file CMI-20-na-s002.tif]

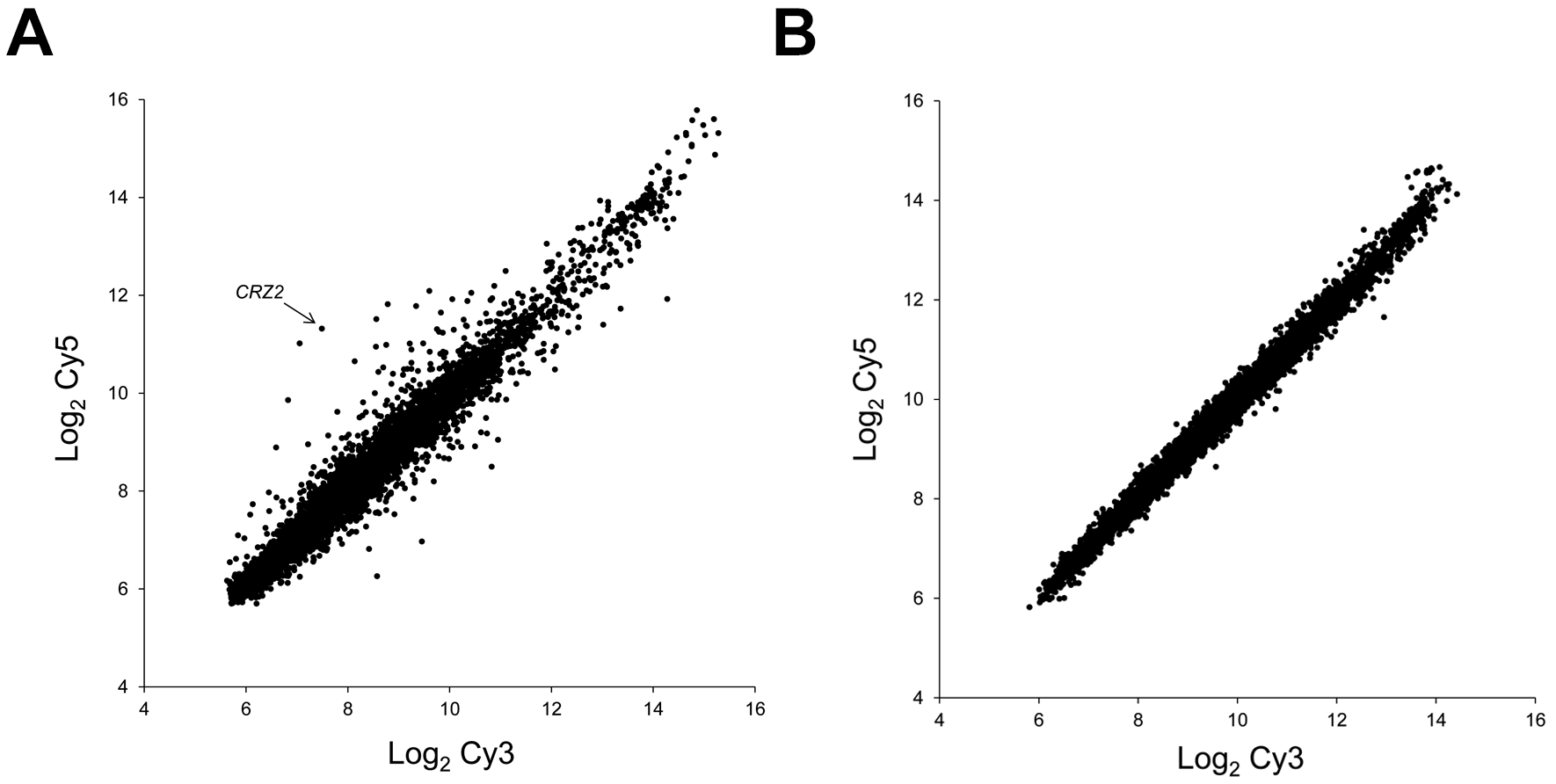

Supplement: Supplementary file 3 — Supporting info item [file CMI-20-na-s003.tif]

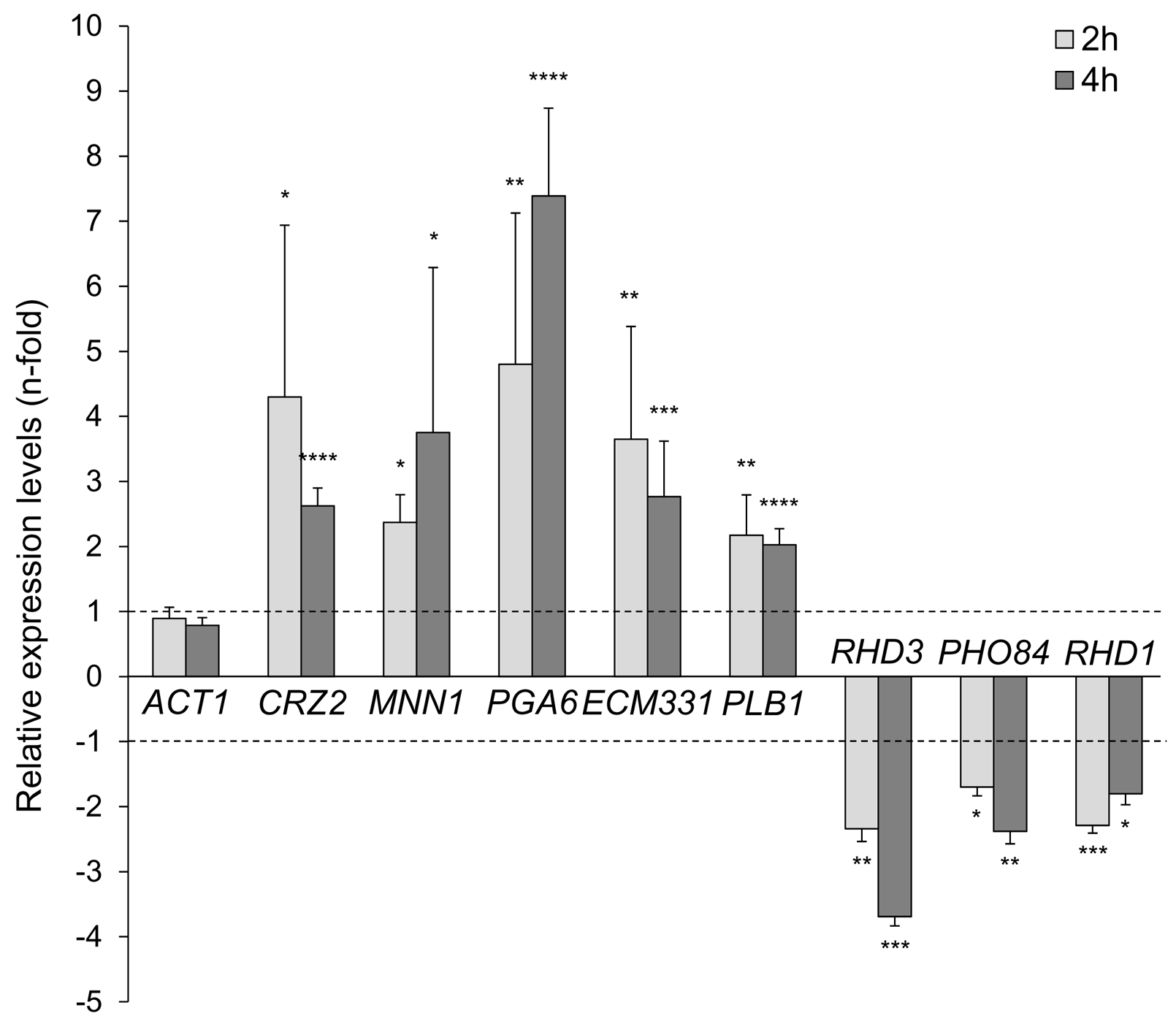

Supplement: Supplementary file 4 — Supporting info item [file CMI-20-na-s004.tif]

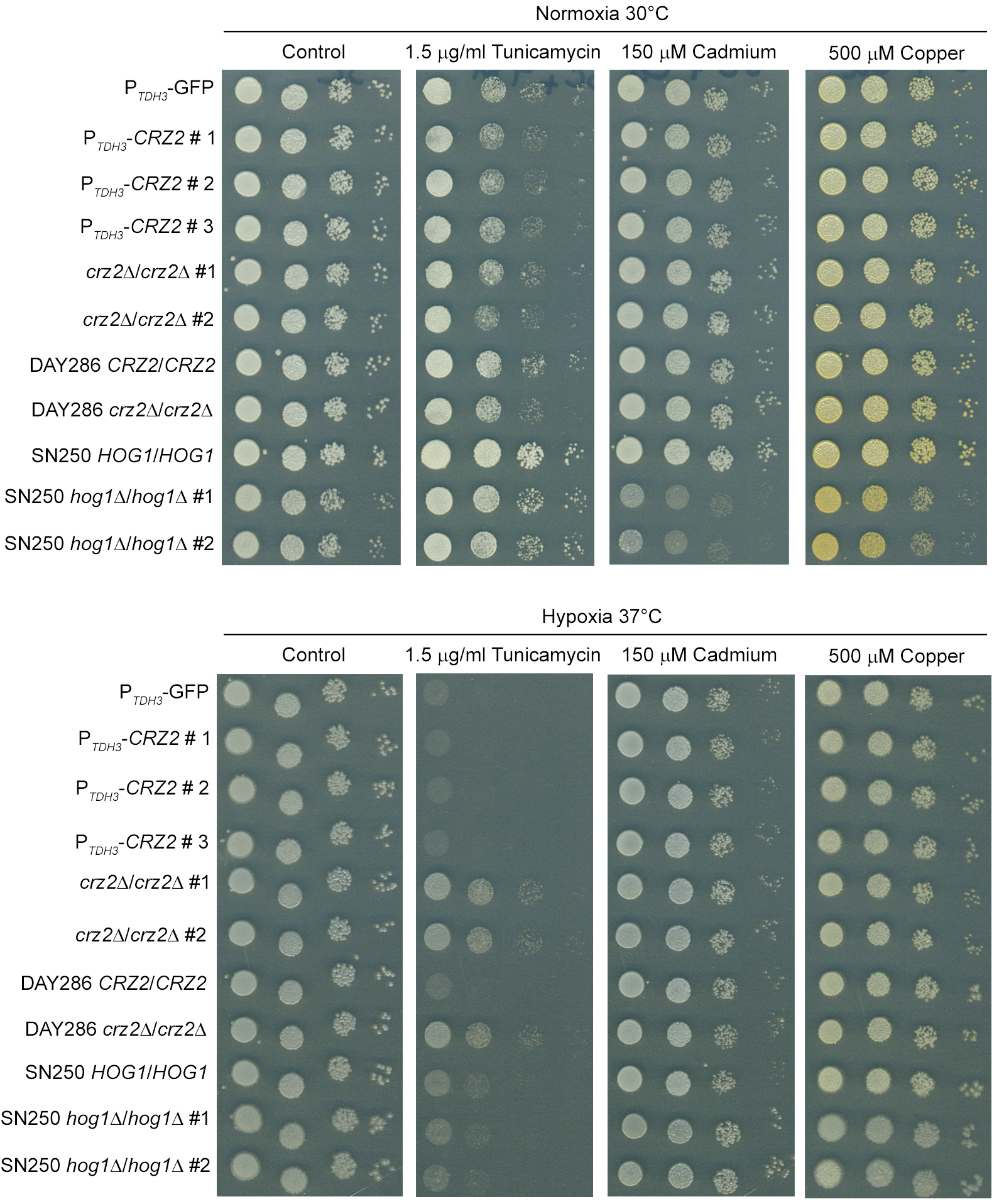

Supplement: Supplementary file 5 — Supporting info item [file CMI-20-na-s005.tif]
